# Cross-species analysis of enhancer logic using deep learning

**DOI:** 10.1101/gr.260844.120

**Published:** 2020-12

**Authors:** Liesbeth Minnoye, Ibrahim Ihsan Taskiran, David Mauduit, Maurizio Fazio, Linde Van Aerschot, Gert Hulselmans, Valerie Christiaens, Samira Makhzami, Monika Seltenhammer, Panagiotis Karras, Aline Primot, Edouard Cadieu, Ellen van Rooijen, Jean-Christophe Marine, Giorgia Egidy, Ghanem-Elias Ghanem, Leonard Zon, Jasper Wouters, Stein Aerts

**Affiliations:** 1 VIB-KU Leuven Center for Brain and Disease Research, 3000 Leuven, Belgium;; 2 KU Leuven, Department of Human Genetics KU Leuven, 3000 Leuven, Belgium;; 3 Howard Hughes Medical Institute, Stem Cell Program and the Division of Pediatric Hematology/Oncology, Boston Children's Hospital and Dana-Farber Cancer Institute, Harvard Medical School, Boston, Massachusetts 02115, USA;; 4 Department of Stem Cell and Regenerative Biology, Harvard Stem Cell Institute, Cambridge, Massachusetts 02138, USA;; 5 Laboratory for Disease Mechanisms in Cancer, KU Leuven, 3000 Leuven, Belgium;; 6 Center for Forensic Medicine, Medical University of Vienna, 1090 Vienna, Austria;; 7 Division of Livestock Sciences (NUWI) - BOKU University of Natural Resources and Life Sciences, 1180 Vienna, Austria;; 8 VIB-KU Leuven Center for Cancer Biology, 3000 Leuven, Belgium;; 9 KU Leuven, Department of Oncology KU Leuven, 3000 Leuven, Belgium;; 10 CNRS-University of Rennes 1, UMR6290, Institute of Genetics and Development of Rennes, Faculty of Medicine, 35000 Rennes, France;; 11 Université Paris-Saclay, INRA, AgroParisTech, GABI, 78350 Jouy-en-Josas, France;; 12 Institut Jules Bordet, Université Libre de Bruxelles, 1000 Brussels, Belgium

## Abstract

Deciphering the genomic regulatory code of enhancers is a key challenge in biology because this code underlies cellular identity. A better understanding of how enhancers work will improve the interpretation of noncoding genome variation and empower the generation of cell type–specific drivers for gene therapy. Here, we explore the combination of deep learning and cross-species chromatin accessibility profiling to build explainable enhancer models. We apply this strategy to decipher the enhancer code in melanoma, a relevant case study owing to the presence of distinct melanoma cell states. We trained and validated a deep learning model, called DeepMEL, using chromatin accessibility data of 26 melanoma samples across six different species. We show the accuracy of DeepMEL predictions on the CAGI5 challenge, where it significantly outperforms existing models on the melanoma enhancer of *IRF4*. Next, we exploit DeepMEL to analyze enhancer architectures and identify accurate transcription factor binding sites for the core regulatory complexes in the two different melanoma states, with distinct roles for each transcription factor, in terms of nucleosome displacement or enhancer activation. Finally, DeepMEL identifies orthologous enhancers across distantly related species, where sequence alignment fails, and the model highlights specific nucleotide substitutions that underlie enhancer turnover. DeepMEL can be used from the Kipoi database to predict and optimize candidate enhancers and to prioritize enhancer mutations. In addition, our computational strategy can be applied to other cancer or normal cell types.

A cell's phenotype arises from the expression of a unique set of genes, which is regulated through the binding of transcription factors (TFs) to *cis*-regulatory regions, such as promoters and enhancers. Deciphering gene regulatory programs entails mapping the network of TFs and *cis*-regulatory regions that govern the identity of a given cell type, as well as understanding how the specificity of such a network is encoded in the DNA sequence of genomic enhancers. Profiling accessible chromatin via DNase I hypersensitive sequencing (DNase-seq) or via the assay for transposase-accessible chromatin using sequencing (ATAC-seq) represents a useful approach for identifying putative enhancers (Song and Crawford 2010; Buenrostro et al. 2013; Klemm et al. 2019). Indeed, active enhancers are typically depleted of one or more nucleosomes owing to the binding of TFs. Initial changes in DNA accessibility can be facilitated through a special class of TFs that bind with high affinity to their recognition sites and that have a long residence time at the enhancer, sometimes referred to as pioneer TFs (Zaret and Carroll 2011; Klemm et al. 2019). By displacing nucleosomes or thermodynamically outcompeting nucleosome binding, they allow other TFs to cobind, thereby further stabilizing the nucleosome-depleted region and/or actively enhancing transcription of target genes (Grossman et al. 2018; Jacobs et al. 2018; Dodonova et al. 2020).

Because the presence and architecture of TF binding sites within enhancers determines which TFs can bind with high affinity, understanding this “enhancer logic” can help interpret the functional role of enhancers within a gene regulatory network. Several techniques exist to study the enhancer code, including (1) motif discovery tools (Bailey et al. 2009; Heinz et al. 2010; Thomas-Chollier et al. 2011, 2012; Janky et al. 2014; Imrichová et al. 2015); (2) comparative genomics (Ballester et al. 2014; Prescott et al. 2015; Villar et al. 2015); (3) genetic screens (Gasperini et al. 2019; Kircher et al. 2019); and (4) machine learning techniques (Park and Kellis 2015). In particular, the latter has seen a strong boost in recent years with the advent of large training sets derived from genome-wide profiling. Three pivotal methods based on deep learning include DeepBind (Alipanahi et al. 2015), DeepSEA (Zhou and Troyanskaya 2015), and Basset (Kelley et al. 2016), the first convolutional neural networks (CNNs) applied to genomics data (Eraslan et al. 2019). Since their emergence in the genomics field, machine learning techniques, and especially CNNs, have been applied to model a range of regulatory aspects, including cross-species enhancer predictions (Min et al. 2016; Quang and Xie 2016; Chen et al. 2018), TF binding sites (Wang et al. 2018; Avsec et al. 2020), DNA methylation (Angermueller et al. 2017), and 3D chromatin architecture (Schreiber et al. 2017).

Deciphering gene regulation and the underlying enhancer code is not only important during dynamic processes such as development, but also in disease contexts such as cancer, where gene regulatory networks are typically misregulated owing to mutations. Particularly in melanoma, a type of skin cancer that develops from melanocytes, gene expression is misregulated and highly plastic (Shain and Bastian 2016; Rambow et al. 2019). This gives rise to two main melanoma cell states: the melanocytic (MEL) state, which still resembles the cell of origin, expressing high levels of the melanocyte-lineage specific transcription factors MITF, SOX10, and TFAP2A, as well as typical pigmentation genes such as *DCT*, *TYR*, *PMEL*, and *MLANA*; and the mesenchymal-like (MES) state, in which the cells are more invasive and therapy resistant, expressing high levels of genes involved in TGFB signaling and epithelial-to-mesenchymal transition (EMT)-related genes (Hoek et al. 2006, 2008; Verfaillie et al. 2015; Rambow et al. 2019; Wouters et al. 2020). These transcriptomic differences have also been studied at the epigenomics level, with AP-1 and TEAD factors as master regulators of the MES state and binding sites for SOX10 and MITF significantly enriched in MEL-specific regulatory regions (Verfaillie et al. 2015; Bravo González-Blas et al. 2019; Wouters et al. 2020). However, it remains unclear how these regulatory states are encoded in particular enhancer architectures and whether such architectures are evolutionary conserved. Besides human cell lines and human patient–derived cultures, several animal models have been established in melanoma research, including mouse, pig, horse, dog, and zebrafish (Egidy et al. 2008; Seltenhammer et al. 2014; van der Weyden et al. 2016; van Rooijen et al. 2017; Segaoula et al. 2018; Prouteau and André 2019). Although these models are widely used, it is unknown whether their enhancer landscapes and regulatory programs are conserved with human. Here, we take advantage of these animal model systems and combine cross-species chromatin accessibility profiling with deep learning, to investigate enhancer logic in melanoma.

## Results

### Melanoma chromatin accessibility landscapes are conserved across species

**Figure 1.**
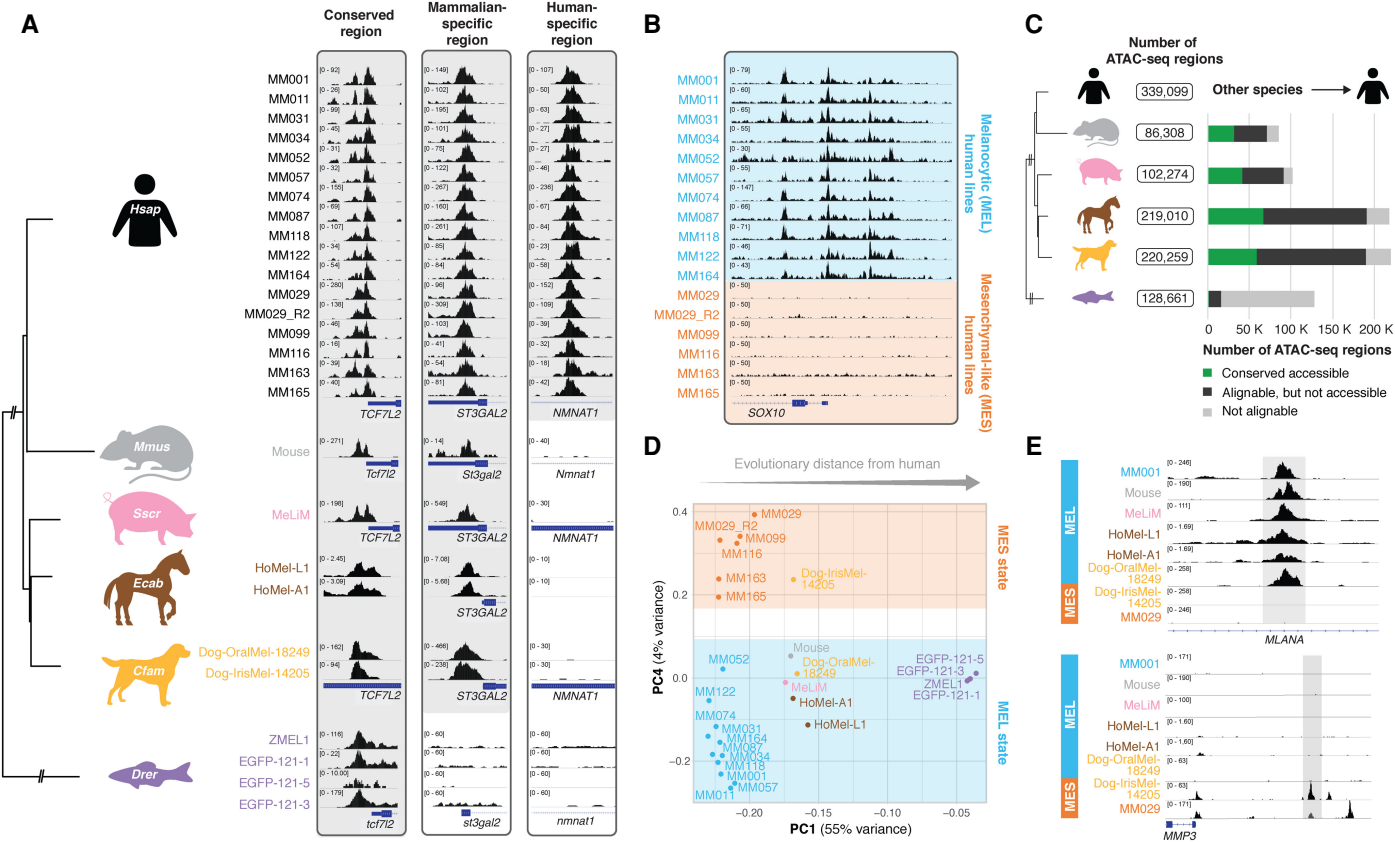
Comparative epigenomics reveals conservation of two main melanoma states. (*A*) Evolutionary relationship between the six studied species, represented by a phylogenetic tree (NCBI taxonomy tree). ATAC-seq profiles of the 26 melanoma cell lines are shown for three regulatory regions. (*B*) ATAC-seq profiles of the human melanoma lines for the *SOX10* locus. Lines are colored by the melanocytic (MEL, in blue) or mesenchymal-like (MES, in orange) melanoma state. (*C*) Total number of ATAC-seq regions observed across all samples of a species are colored based on whether they are not alignable, alignable, or conserved accessible in human. (*D*) PCA clustering based on the accessibility of the 29,619 alignable regions across all six species. (*E*) ATAC-seq profiles of MEL and MES lines of different species for an intronic *MLANA* enhancer and the upstream region of *MMP3*.

We profiled chromatin accessibility using ATAC-seq on a collection of melanoma cell lines across six species, for a total of 26 samples (Fig. 1A). These include 16 human patient–derived cultures (MM lines) (Gembarska et al. 2012; Verfaillie et al. 2015), one mouse cell line (Dankort et al. 2009), primary melanoma cells from the pig melanoma model MeLiM (MeLiM) (Egidy et al. 2008), two horse melanoma lines derived from a Grey Lipizzaner horse (HoMel-L1) and from an Arabian horse (HoMel-A1) (Seltenhammer et al. 2014), two dog melanoma cell lines from oral and uveal sites (Dog-OralMel-18249 and Dog-IrisMel-14205, respectively; Cani-DNA BRC: https://dog-genetics.genouest.org), and four melanoma lines established from zebrafish (ZMEL1, EGFP-121-1, EGFP-121-5, and EGFP-121-3) (White et al. 2008, 2011). Per sample, between 65,475 and 176,695 ATAC-seq peaks were called, with distinct levels of conservation of accessibility across the species (Fig. 1A; Supplemental Fig. S1A). The difference in the number of peaks across the samples is attributable, on the one hand, to genome size (Supplemental Fig. S1B), and on the other hand, to data quality (measured as the fraction of reads in peaks [FRiP]) (Supplemental Fig. S1C).

Unsupervised clustering of the 16 human lines revealed two distinct groups (Supplemental Fig. S1D), which correspond to the two main cell states in human melanoma, that is, the melanocytic state (MEL) and mesenchymal-like state (MES), as was further confirmed for most of the cell lines by previously generated RNA-seq data (Supplemental Fig. S1E; Verfaillie et al. 2015) and corroborated by previous studies using epigenomics data (Verfaillie et al. 2015; Wouters et al. 2020). Indeed, regulatory regions near MEL-specific genes such as *SOX10* are accessible in human lines in the MEL state (MM001, MM011, MM031, MM034, MM052, MM057, MM074, MM087, MM118, MM122, and MM164), whereas they are closed in MES melanoma lines (MM029, MM099, MM116, MM163, and MM165) (Fig. 1B). As in Wouters et al. (2020), we observed heterogeneity between samples of the MEL state (Supplemental Fig. S1D).

To enable the comparison of chromatin accessibility between human and other species, we first identified regulatory regions that are alignable (i.e., have a high sequence similarity) between species using the liftOver tool (at least 10% of bases must remap) (Meyer et al. 2012). When such an alignable region contains an ATAC-seq peak in the compared species, it is referred to as a “conserved accessible” region. Between 1.1% and 40.9% of the ATAC-seq regions in non-human lines were conserved accessible in human (Fig. 1C), and between 0.9% and 18.4% of the human peaks were conserved accessible in the other species (Supplemental Fig. S1F). Accordingly, we identified 303,392 alignable and 10,592 conserved accessible regions across all mammalian species. This number decreases when including zebrafish, to 29,619 alignable regions and, only 116 conserved accessible regions. Nearly half of the 10,592 conserved accessible mammalian regions were promoters within 1 kb of a transcription start site (Supplemental Fig. S1G). Indeed, high conservation of proximal promoters has previously been reported (Villar et al. 2015). In each of the mammalian species, the 10,592 conserved accessible regions were more accessible compared to all ATAC-seq regions; in addition, they show a higher ChIP-seq signal for acetylation of histone H3 at lysine 27 (H3K27ac) in human, a mark for active regulatory regions (Supplemental Fig. S1H,I; Creyghton et al. 2010), and higher sequence conservation compared to alignable regions as measured by phastCons and phyloP (Supplemental Fig. S1J; Siepel 2005; Pollard et al. 2010). Nevertheless, although ATAC-seq regions are nucleosome depleted and often bound by several TFs, they are not necessarily active enhancers, because accessibility does not directly translate to enhancer activity (Shlyueva et al. 2014).

Next, we examined whether the MEL and MES melanoma states are conserved in the other species of our cohort. Clustering all mammalian samples based on the accessibility of the 303,392 alignable regions (Supplemental Fig. S1K), or of all samples (including zebrafish) using the 29,619 alignable regions (Fig. 1D), revealed two axes of variation between the samples, namely (1) the evolutionary variation between species and (2) the distinction between the melanoma states. All human MEL samples are clustered together with nine of the 10 non-human lines, indicating that most of the non-human cell lines are epigenomically similar to the human MEL lines. Conversely, the dog cell line Dog-IrisMel-14205 clustered together with the human MES samples, which indicates that Dog-IrisMel-14205 belongs to the MES state. This classification of melanoma samples was reflected in their accessibility at known MEL and MES regulatory regions such as the intronic enhancer of *MLANA,* a MEL-specific gene involved in melanosome biogenesis (De Mazière et al. 2002), and an enhancer upstream of *MMP3*, a gene that increases metastatic potential in melanoma cell lines (Fig. 1E; Shoshan et al. 2016). Classifying the cross-species samples based on a principal component analysis (PCA) of only the conserved accessible regions (i.e., without species-specific or clade-specific peaks) clearly revealed the MEL-MES distinction, whereas the species variation was less outspoken (Supplemental Fig. S1L,M).

In conclusion, by using ATAC-seq on a panel of 26 melanoma lines across six species, conserved accessible regulatory regions could be identified. These regions allowed clustering of the melanoma samples into two groups that correspond to the two main melanoma cell states, indicating conservation of the MES melanoma state in dog and the MEL melanoma state in pig, mouse, horse, dog, and even zebrafish melanoma samples.

### Conservation of transcription factor motifs in state-specific enhancers

**Figure 2.**
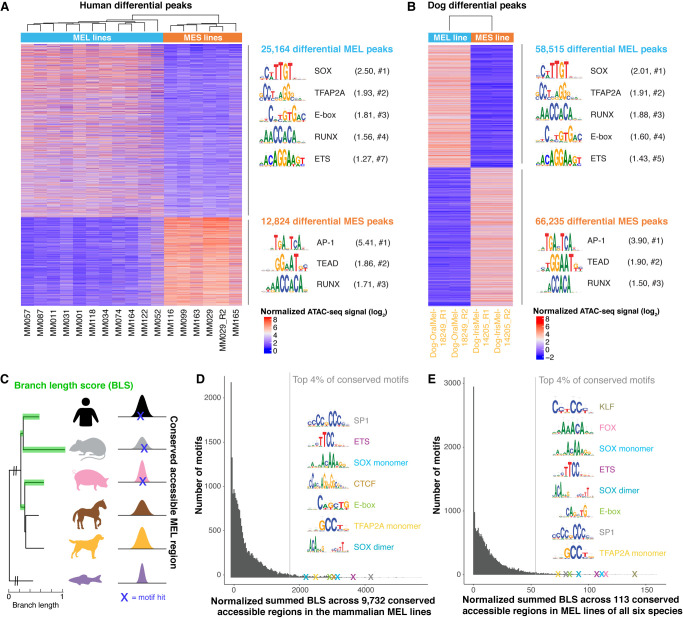
Conservation of binding motifs of master regulators of MEL and MES melanoma states. (*A*,*B*) Heatmap of differential ATAC-seq regions when comparing human MEL versus human MES lines (*A*) and the MEL dog line “Dog-OralMel-18249” versus the MES dog line “Dog-IrisMel-14205” (two biological replicates each) (*B*), colored by normalized ATAC-seq signal. Enriched TF binding motifs in the differential peaks were identified via HOMER (Heinz et al. 2010), and the first logo of enriched TF families is shown. The ratio of the percentage of target and background sequences with the motif is indicated between brackets, as well as the rank of the TF class within the HOMER output (#). (*C*) Schematic overview of cross-species motif analysis using the branch length score (BLS) as a measure for the evolutionary conservation of a motif hit across conserved accessible regions. The BLS was summed across a set of conserved accessible regions. (*D*,*E*) Histogram of the normalized summed BLS score for 20,003 motifs on 9732 conserved accessible regions across the mammalian MEL lines (*D*) and on 113 conserved accessible regions across MEL lines of all six species (*E*). The first hit of the top recurrent TF binding motifs within the top 4% conserved motifs is indicated as a cross and is accompanied by the logo of the motif.

Next, we investigated whether TF binding motifs that are specific to the MEL and MES states are conserved across species. To this end, we performed differential motif enrichment between MEL and MES accessible regions for human and dog, because these were the two species in our cohort for which cell lines of both states were identified above. Differential peak calling (log_2_FC > 2.5 and *P*_Adj_ < 0.0005), followed by motif enrichment using HOMER (Heinz et al. 2010), revealed a highly similar enrichment of SOX, TFAP2 family, E-box, RUNX, and ETS TF binding motifs in both the human and dog MEL-specific peaks (Fig. 2A,B; for complete HOMER output, see Supplemental Table S1). The enriched motifs of the TFAP2 family can most likely be linked to TFAP2A because this is a master regulator in human melanocytes and melanoma (Seberg et al. 2017). Similarly, the observed E-box and SOX motifs most likely represent MITF and SOX10, respectively, because they are among the previously reported master regulators in human MEL lines (Hoek et al. 2006; Verfaillie et al. 2015; Bravo González-Blas et al. 2019; Wouters et al. 2020). Likewise, motif enrichment in the MES regions is very similar between human and dog, revealing AP-1 and TEAD motifs as most highly enriched (Fig. 2A,B), corroborating earlier findings (Verfaillie et al. 2015). Together, these observations indicate that the MEL and MES melanoma cell states are conserved in dog and that they are likely governed by the same master regulators, based on the concordance of motif enrichment.

To further verify the importance of the MEL-specific master regulators in MEL cell lines of the remaining four species, we applied a different strategy because we could not contrast MEL and MES lines for horse, pig, mouse, and zebrafish. We analyzed 9732 accessible regions that are conserved accessible across all mammalian MEL lines to identify conserved TF binding sites. We scanned these regions using the cisTarget motif collection (v8) (Herrmann et al. 2012; Janky et al. 2014; Imrichová et al. 2015) containing 20,003 TF position-weight matrices (PWMs) and used a branch length score (BLS) to calculate the level of evolutionary conservation of each TF binding motif (Fig. 2C), a strategy applied before in other systems (Stark et al. 2007; Jacobs et al. 2018). Among the 4% most conserved motifs were SP1, ETS, SOX, CTCF, MITF, and TFAP2A motifs (Fig. 2D). The top conserved motifs were members of the SP/KLF TF family, which bind to GC-rich motifs in promoters (Dynan and Tjian 1983). Indeed, 47% of the 9732 conserved accessible regions in mammalian MEL lines are proximal promoters (≤1 kbp from TSS). BLS scoring on the remaining 5196 more distal conserved accessible regions revealed similar highly conserved motifs, except for SP/KLF TF family motifs, indicating that distal regions, such as enhancers, mostly contain the state-specific TF binding motifs (Supplemental Fig. S1N). In the 113 conserved accessible regions across the MEL cell lines across all six species, BLS scoring again revealed SOX, ETS, MITF, and TFAP2A motifs among the most conserved motifs (Fig. 2E).

In conclusion, two independent strategies of motif analysis suggest conservation of TF binding sites for known melanoma master regulators, with conserved SOX10, MITF, TFAP2A, and ETS TF family motif enrichment in MEL enhancers across all six studied species.

### Deep neural network DeepMEL reveals nucleotide-resolution enhancer logic

**Figure 3.**
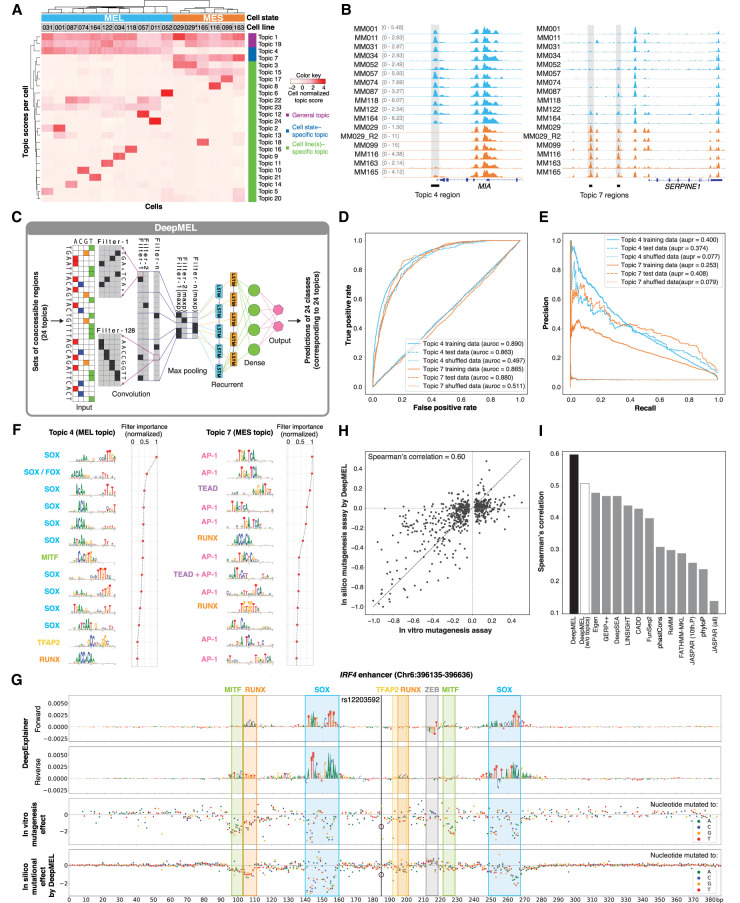
DeepMEL classifies melanoma enhancers and predicts important TF binding motifs. (*A*) Cell-topic heatmap of cisTopic applied to 339,099 ATAC-seq regions across the 16 human melanoma lines, colored by normalized topic scores. (029*) MM029_R2. (*B*) Example regions of a MEL-specific (topic 4) region near *MIA* and MES-specific (topic 7) regions upstream of *SERPINE1*. (*C*) Schematic overview of DeepMEL. Twenty-four topics or sets of coaccessible regions were used as input for training of a multiclass multilabel neural network. (*D,E*) Receiver operating characteristic curve (*D*) and precision recall curve (*E*) for DeepMEL on training, test, and shuffled data of topic 4 and topic 7 regions. (*F*) Top enriched filters learned by DeepMEL to classify regions as MEL (topic 4) or MES (topic 7). Normalized filter importance is shown per filter. (*G*) Example of a MEL-predicted enhancer near *IRF4*. (First and second rows) DeepExplainer view of the forward and reverse strand, with the height of the nucleotides indicating the importance for prediction of the MEL enhancer. (Third row) In vitro effect of point mutations on enhancer activity as measured by MPRA (Kircher et al. 2019). Colors represent the nucleotide to which the wild-type nucleotide is mutated. (Fourth row) In silico effect of point mutations as predicted by DeepMEL. (*H*) Correlation between the in vitro mutational effects on the *IRF4* enhancer and the in silico mutagenesis predictions. (*I*) Performance of variant effect prediction of DeepMEL using topics (black bar, model used in this paper) or using ATAC-seq signal (white bar), and several previously tested models on the *IRF4* enhancer case (Kircher et al. 2019).

Although motif enrichment can predict candidate regulators, we sought to build a more comprehensive model of the MEL enhancers, which would allow cross-species predictions and in-depth analysis of enhancer architecture. To this end, we trained a deep learning (DL) model on the human ATAC-seq data. First, to construct an unsupervised training set, we clustered all 339,099 human ATAC-seq peaks using cisTopic—a probabilistic framework to analyze scATAC-seq data that can also be applied to bootstrapped bulk ATAC-seq data (Bravo González-Blas et al. 2019; Methods)—into 24 “topics” or sets of coaccessible regions (Fig. 3A; Supplemental Fig. S2A,B). This provided a nuanced classification, with topic 4 and topic 7 representing the MEL- and MES-specific enhancers, respectively, being accessible across all MEL or MES samples (Fig. 3A; Supplemental Fig. S2C). In addition, we found two topics with regions that are generally accessible across all cell lines (topic 1 and topic 19) (Fig. 3A; Supplemental Fig. S2C). These ubiquitously accessible regions are highly enriched for proximal promoters (Supplemental Fig. S2D) and for known promoter-specific TF binding motifs linked to SP and NFY TF families (Supplemental Fig. S2C; Dynan and Tjian 1983; Maity and de Crombrugghe 1998). Other topics were more specific to one or a small group of cell lines (Fig. 3A). We verified the biological relevance of these topics by Gene Ontology (GO) enrichment of flanking genes using GREAT (McLean et al. 2010). Genes near topic 4 regions are significantly enriched for GO terms such as pigmentation (FDR = 1.95 × 10^−8^) and neural crest cell differentiation (FDR = 4.26 × 10^−7^), whereas genes near topic 7 regions were enriched for GO terms involved in cell–cell adhesion (1.56 × 10^−13^). Motif discovery on the top regions assigned to each topic confirmed enrichment of SOX, ETS, TFAP2A, and MITF motifs in the MEL topic regions (topic 4) and AP-1 in the MES topic (topic 7) (Supplemental Fig. S2C). An example topic 4 region in the promoter of the SOX10 target gene *MIA* (Graf et al. 2014) is shown in Figure 3B, as well as two topic 7 regions upstream of *SERPINE1,* a gene expressed in metastatic melanoma (Klein et al. 2012).

Using the 24 topics as classes, we trained a multiclass, multilabel classifier using a neural network, called “DeepMEL” (Fig. 3C). As input, we used the forward and reverse complement of 500-bp sequences centered on the ATAC-seq summit. As topology, we used the DanQ CNN-RNN hybrid architecture (Quang and Xie 2016) consisting of four main layers: a convolution layer to discover local patterns in sequential data, followed by a max-pooling layer to reduce the dimensionality of the data and generalize the model effectively, a bidirectional recurrent layer (LSTM) to detect long-range dependencies of the local patterns discovered in the first layer, and finally a fully connected (dense) layer just before the output layer to help the classification after the feature extraction layers (Fig. 3C). Note that several hyperparameters, including the number and size of the convolutional filters and the length of the input DNA sequence, were optimized to yield the final model (Supplemental Fig. S3; Supplemental Note). After successful training of DeepMEL—area under the receiver operating characteristic curve (auROC) = 0.863 and area under the precision recall curve (auPR) = 0.374 on test data for topic 4 regions (Fig. 3D,E; Supplemental Fig. S4A)—we used the weights of the neurons from the convolutional filters to extract local patterns learned by the model. We transformed these convolution filters into PWMs and found the importance of each filter for each topic (Methods). Filters that represent SOX, MITF, TFAP2A, and RUNX motifs were most relevant for the MEL-specific topic 4; filters that represent AP-1, TEAD, and RUNX binding sites were assigned to the MES-specific topic 7 (Fig. 3F). Thus, DeepMEL learned the relevant features de novo from the sequence. The 3885 regions classified as MEL-specific in MM001 (topic 4 scores above threshold of 0.16) (Methods) were not only highly accessible in MEL lines and closed in MES lines (Supplemental Fig. S4B), but were also accessible in human melanocytes (Supplemental Fig. S4C), indicating that MEL-specific melanoma regions are not cancer-specific but already accessible in their cell of origin, that is, the melanocytes. As a consequence, we can potentially extrapolate the observations on this topic to normal melanocyte enhancers. Although in the remainder of this work we will score accessible regions to identify functional enhancers, it is also possible to score the entire genome, without filtering for ATAC-seq peaks (Supplemental Fig. S4D).

To examine the TF binding site architecture within enhancers, we used a model interpretation tool, DeepExplainer (Lundberg and Lee 2017; Avsec et al. 2020; Lundberg et al. 2020). For a MEL enhancer located on the fourth intron of *IRF4,* nucleotides important for classifying this enhancer as topic 4 emerge as motifs for SOX10, MITF, TFAP2A, and RUNX factors (Fig. 3G, top two rows; for another example, see Supplemental Fig. S4E,F).

It is known that enhancer accessibility does not directly translate to enhancer activity (Shlyueva et al. 2014). To test whether the same TF binding motifs contribute to the activity of MEL enhancers, we used the *IRF4* enhancer as case study. For this enhancer, Kircher et al. (2019) performed saturation mutagenesis followed by an in vitro massively parallel reporter assay (MPRA), testing the effect of every possible single-nucleotide mutation on enhancer activity (Fig. 3G, third row). The most deleterious mutations coincided with the DeepMEL-predicted SOX, E-box, and RUNX-like motifs, overlapping with nucleotides that also have the strongest in silico effect (Fig. 3G, last row), indicating that the predicted motifs are actually contributing to enhancer activity. In addition, the magnitude of the in silico predicted effect highly correlates with the effect of the in vitro mutations (Spearman's correlation of 0.60) (Fig. 3G,H). These observations indicate that, although DeepMEL was trained to predict binary enhancer accessibility, it is also a good predictor of enhancer activity of this specific enhancer. DeepMEL predictions outperform other classifiers and deep learning models that were benchmarked in Kircher et al. (2019) (Fig. 3I). One possible explanation for this improvement is that DeepMEL uses more nuanced topics (Fig. 3I, black bar) rather than the ATAC-seq signal of the different MM lines as labels (Fig. 3I, white bar). Enhancer accessibility and activity cannot only be influenced by mutations that break a motif for an activating TF, but also by the creation of a repressor binding motif, as was, for instance, the case for the SNP rs12203592 (Fig. 3G; Supplemental Fig. S4G).

In conclusion, DeepMEL, trained on topics of human coaccessible regions, is performant in classifying melanoma regulatory regions into different classes based on purely the DNA sequence. Features learned by DeepMEL correspond to TF binding motifs of master regulators of specific classes. These motifs can also be located and visualized within regions using a model interpretation tool, allowing examination of the motif architecture within specific enhancers, and predicting the effect of mutations on enhancer accessibility.

### Cross-species scoring identifies orthologous melanoma enhancers

**Figure 4.**
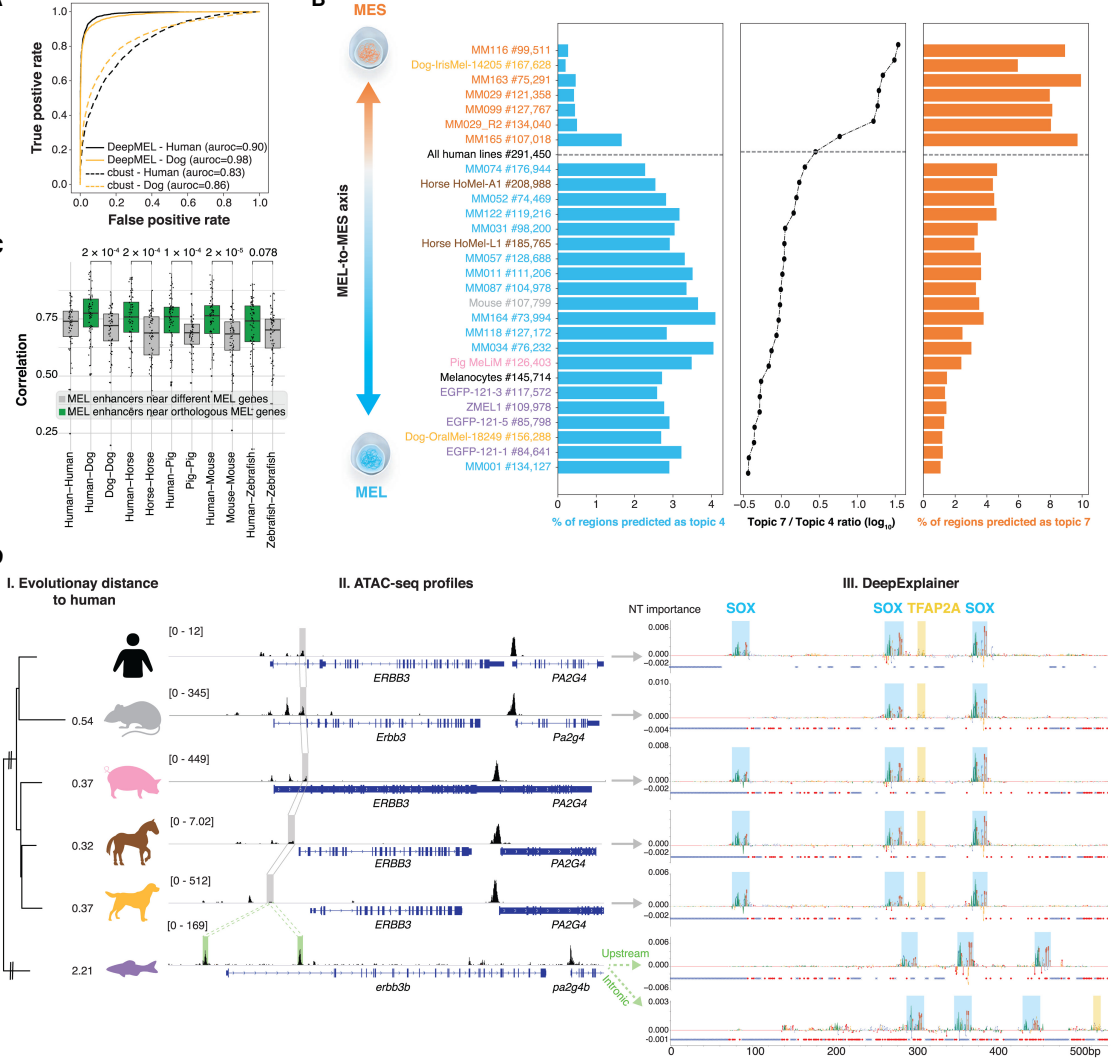
Human-trained deep learning model applied to cross-species ATAC-seq data. (*A*) Performance of DeepMEL and Cluster-Buster (cbust) in classifying MEL and MES differential peaks in human and dog. (*B*) Percentage of MEL- and MES-predicted ATAC-seq regions across all samples in our cohort and in human melanocytes. Samples are ordered according to the ratio of the number of MES/MEL-predicted regions. (*C*) Pearson's correlation of deep layer scores between MEL-predicted regions near orthologous MEL genes between human and another species (Human-Species) or between MEL-predicted regions near different MEL genes within one species (Species-Species). *P*-values of unpaired two-sample Wilcoxon tests are reported. (*D*) (I) Evolutionary distance between human and other species in branch length units. (II) ATAC-seq profiles of the *ERBB3* locus in the six species. MEL-specific enhancers that were predicted by DeepMEL and that were also found (gray) or not found (green) via liftOver of the human MEL enhancer are highlighted. (III) DeepExplainer plots for the multiple-aligned MEL-predicted *ERBB3* enhancers. Red and blue dots represent point and indel mutations, respectively.

Next, we asked whether the human-trained model DeepMEL can be used to predict MEL and MES enhancers in other species. We started with the dog genome as a test case, because the differential ATAC-seq peaks between the MEL (Dog-OralMel-18249) and MES (Dog-IrisMel-14205) dog cell lines can serve as true positives (Fig. 4A). DeepMEL reached similar performance in human and dog for predicting MEL and MES regions, and this accuracy is significantly higher compared to using *cis*-regulatory module (CRM) scoring with PWMs (Fig. 4A). Having confirmed that the human model can identify enhancers in the dog genome, we predicted MEL and MES enhancers across all six species. This furthermore allowed us to order all samples according to the MEL-MES axis (Fig. 4B). Between 2093 and 5400 MEL enhancers were predicted, and between 7459 and 10,743 MES enhancers, in samples of the MEL and MES state, respectively (Fig. 4B). The majority of these enhancers could not have been detected using whole-genome alignments (liftOver) (Supplemental Fig. S5A–E). Of note, predicted MEL enhancers in the pig melanoma cells (MeLiM) were similarly accessible in pig melanocytes (Supplemental Fig. S5F), again indicating that MEL melanoma enhancers can be used as a model for melanocyte enhancers.

Next, we compared the occurrence of MEL enhancers between species in relation to putative target genes. Particularly, we looked at enhancers located near a set of 379 human genes that are specifically expressed in the MEL state (Methods). Of these 379 genes, 217 (67%) had at least one MEL-predicted enhancer within 200 kb upstream of and downstream from the gene. Between 70% and 85% of the orthologous MEL genes in other species had at least one MEL enhancer 200 kb upstream of and downstream from the gene (Supplemental Fig. S5G). Only a small subset of these enhancers could have been found using liftOver (2%–43%, depending on the species). Of these genes, 32 form a core set of conserved MEL-specific genes throughout all species including zebrafish, each having a MEL enhancer nearby. Examples of genes in the core set are *MITF*, *PMEL*, and *TYRP1*, genes known to be involved in melanocyte development, melanosome formation, and melanin production (D'Mello et al. 2016).

A long-standing question in enhancer studies is how to compare enhancers with each other, if their sequences do not align (Cliften et al. 2001; Arunachalam et al. 2010). Here, we tackle this question by using the dense layer of DeepMEL as a reduced dimensional space to calculate the correlation between enhancers. Using this measure we found that MEL-predicted enhancers in proximity of orthologous MEL genes are significantly more similar to each other compared to both MEL-predicted enhancers in proximity of different MEL genes within the same species (Fig. 4C), and redundant (or shadow) (Hong et al. 2008) enhancers linked to the same MEL gene in a species, as well as random non-MEL ATAC-seq peaks near homologous MEL genes (Supplemental Fig. S5H). This altogether supports the idea that MEL enhancers near orthologous genes are indeed orthologous enhancers.

Last, we studied an example of a MEL enhancer in more detail, namely the enhancer near *ERBB3*. DeepMEL predicts a MEL enhancer upstream or intronic of *ERBB3* in each of the mammalian species, which were also found by liftOver of the human *ERBB3* enhancer (Fig. 4D, II). However, in the zebrafish genome, liftOver was unable to identify the homologous region, whereas DeepMEL predicted two MEL enhancers, one upstream of the TSS of *erbb3b* and another in the first intron. Both zebrafish enhancers were highly correlated with the human *ERBB3* enhancer (deep layer Pearson's correlation of 0.812 and 0.797 for the upstream and intronic zebrafish enhancer, respectively), suggesting that both enhancers are orthologous to the human *ERBB3* enhancer. Applying DeepExplainer to the multiple-aligned sequences revealed a conserved motif architecture in the orthologous mammalian *ERBB3* enhancers containing each three SOX motifs and one TFAP2A motif (Fig. 4D, III). In mouse, one SOX binding site was lost, and mouse is also the mammalian species that is most distant from human, among the included mammals in this study (Fig. 4D, I). The two zebrafish enhancers have a highly similar motif architecture, suggesting that they arose by duplication from a common ancestor enhancer.

In conclusion, we showed that DeepMEL is able to identify MEL- and MES-specific enhancers in different species, which allows studying evolutionary events and enhancer logic within orthologous enhancers, even in distant species such as zebrafish.

### Motif architecture of the MEL enhancer

**Figure 5.**
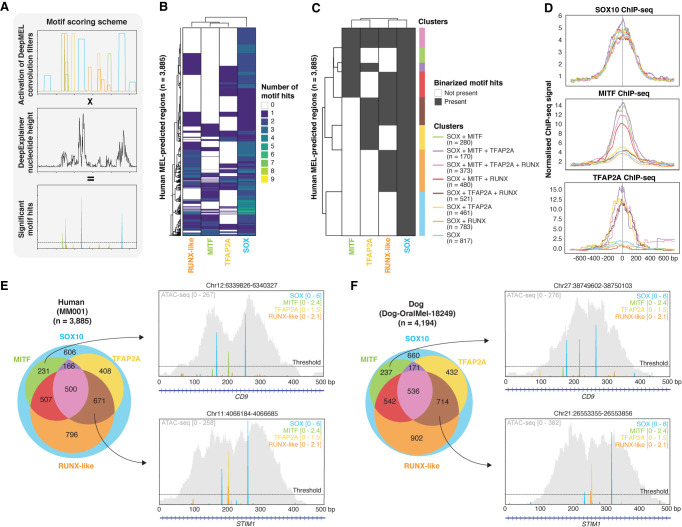
Core Regulatory Complex of MEL melanoma enhancers. (*A*) Schematic overview of motif scoring method in which extended convolutional filter hits from DeepMEL are multiplied by DeepExplainer profiles to yield significant motif hits. (*B*,*C*) Heatmap (*B*) and binarized heatmap (*C*) of the number of significant SOX, TFAP2A, MITF, and RUNX-like motif hits on the 3885 MEL-predicted regions in the human cell line MM001. (*D*) Aggregation plot of normalized ChIP-seq signal of SOX10, MITF, and TFAP2A on the human enhancer clusters. (*E*,*F*) Venn diagram of regions clusters on the 3885 MEL-predicted regions in human (in MM001) (*E*) and the 4194 MEL-predicted regions in dog (in Dog-OralMel-18249) (*F*). Example MEL-predicted enhancers in human and dog are shown for two of the region clusters. The ATAC-seq signal of the regions is shown in gray.

To study the architecture of MEL enhancers in more detail, including motif composition, motif order and distance, and relationships to the position of nucleosomes, we set out to obtain high-confidence motif annotations in each of the 3885 MEL enhancers in human (MM001, the most MEL-like human cell line), for each of the predicted core regulatory factors (SOX10, MITF, TFAP2A, RUNX). To achieve this, we devised an optimized motif scoring method that obtains precise positions of TF binding motifs by multiplying DeepMEL activation scores of convolutional filters (i.e., motifs) with the DeepExplainer profile of each enhancer (Fig. 5A; Methods; Shrikumar et al. 2019).

The first observation was that each MEL enhancer contains at least one SOX10 motif hit, and often two or more (Fig. 5B). This suggests that SOX10 plays a central role in MEL enhancer accessibility. Indeed, knockdown (KD) of SOX10 in MM001 significantly decreases the accessibility of MEL enhancers (Supplemental Fig. S6A), and the regions that close after SOX10-KD are highly enriched for SOX motifs (NES = 28.5), possibly revealing a pioneering-role of SOX10 in MEL enhancers. Next to SOX motifs, a combination of one or multiple TFAP2A, MITF, or RUNX-like motif hits were present in 84% of the MEL-predicted enhancers (Fig. 5B). Next, to facilitate a systematic study of the MEL enhancer logic, we binarized the motif-region matrix to simplify the region clustering (Fig. 5C). We obtained eight different enhancer classes, each with a different motif composition (Fig. 5C). As validation of the clusters and the predicted TF binding sites, we used human ChIP-seq data of SOX10, MITF, and TFAP2A in melanoma or melanocytes (Fig. 5D; Laurette et al. 2015; Seberg et al. 2017). All clusters were indeed highly bound by SOX10, validating the prevalence of the SOX10 motif in MEL enhancers. In contrast, MITF and TFAP2A ChIP-seq data revealed that MITF and TFAP2A bind, respectively, more to enhancers with MITF and TFAP2A sites compared to regions without a predicted MITF or TFAP2A site. These observations indicate that the MEL enhancer architecture does not entail indirect DNA binding of the core regulatory factors because MITF and TFAP2A are only bound when their motifs are present within the enhancer. We further observed that regions containing a TFAP2A site, next to the SOX10 site(s) and possible others, showed a modest increase in accessibility (Supplemental Fig. S6B), which could be in line with the previously described role of TFAP2A as a stabilizer of nucleosome-depleted regions (Grossman et al. 2018). The opposite was true for regions containing RUNX-like binding sites (Supplemental Fig. S6B), suggesting a repressive role of RUNX factors. The presence of a MITF site did not seem to alter the accessibility of enhancers compared to SOX-only enhancers but did increase H3K27ac signal (Supplemental Fig. S6C), possibly indicating that MEL enhancers bound by MITF are more active.

To validate these MEL enhancer classes in other species, we applied the same motif scoring and binarization to DeepMEL-predicted MEL regions in the other species in our cohort. MEL enhancers in other species also clustered into the same eight clusters, with a similar distribution of regions per cluster (Fig. 5E,F; Supplemental Fig. S6D). In addition, liftOver of the clusters showed that the regions of a human cluster correspond more to the same cluster in the other species (Supplemental Fig. S6E), indicating conservation of the MEL enhancer clusters across species. For instance, the dog orthologs of two human MEL enhancers belonging to either the [SOX10 + MITF] cluster (intronic enhancer of *CD9*) or to the cluster containing [SOX10 + TFAP2A + RUNX] (intronic enhancer of *STIM1*) (Fig. 5E) were part of the corresponding clusters in dog (Fig. 5F).

Altogether, these data suggest a Core Regulatory Complex (CoRC) (Arendt et al. 2016) of SOX10, TFAP2A, MITF, and RUNX factors in regulating melanoma MEL enhancers, encoded by a mixed enhancer model (Long et al. 2016), with high flexibility in the combination of binding sites for these four TFs, but with some rigidity (or hierarchy) in the code as at least one SOX10 dimer site is required.

### Putative roles of SOX10 as a pioneer and TFAP2A as a stabilizer in melanoma MEL enhancers

**Figure 6.**
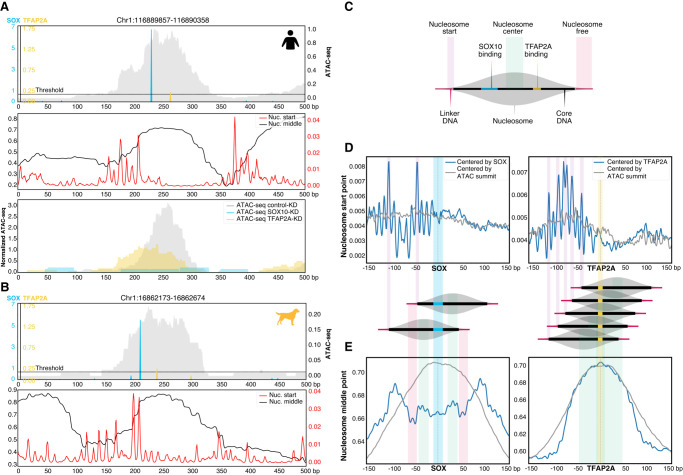
Positional specificity of SOX10 and TFAP2A in MEL melanoma enhancers. (*A*,*B*, *top*) Example human (*A*) and dog (*B*) MEL-predicted enhancer containing significant SOX10 and TFAP2A motifs. The ATAC-seq signal is shown in gray. (*A*, *middle*; *B*, *bottom*) Imputed nucleosome start and middle point profiles. (*A*, *bottom*) For the human example region, ATAC-seq profiles of MM001 in control condition, after 72 h of SOX10 knockdown or TFAP2A knockdown are shown. (*C*) Schematic overview of the nucleosome structure explaining the colors used in *D* and *E*. (*D*,*E*) Nucleosome start point (*D*) and nucleosome middle point predictions (*E*) on MEL-predicted regions containing one SOX10 (*left*) or one TFAP2A motif (*right*) next to possible other motifs, where the regions are either centered on the ATAC-seq summit (gray) or on the SOX10 or TFAP2A motif (blue).

Because previous results suggested a pioneering and stabilizer function for SOX10 and TFAP2A, respectively, we wanted to further investigate these putative roles and how they are mechanistically affecting chromatin accessibility. First, we analyzed the location of binding sites relative to the position of the nucleosome, focusing on a human and dog MEL enhancer that contain a combination of one SOX10 and one TFAP2A site (Fig. 6A,B). We predicted the nucleosome start and middle point using a previously published model (Kaplan et al. 2009) and observed that SOX10 binding sites are situated within the borders of the nucleosome, near the nucleosome start point, whereas TFAP2A binding occurs preferentially near the center of the nucleosome (Fig. 6A,B). KD of TFAP2A halved the accessibility of this specific human region, whereas SOX10-KD completely abolished the ATAC-seq peak (Fig. 6A), indicating that SOX10 is necessary for accessibility, and that TFAP2A further increases the accessibility, which is in line with our previous observations (Supplemental Fig. S6A,B).

These example enhancers raised an interesting positional preference of SOX10 and TFAP2A. To assess whether this occurs globally, we centered human MEL enhancers on the SOX10 and TFAP2A motif hits and calculated the aggregated location of the nucleosome start and middle point (Fig. 6C–E). SOX10 shows a consistent preference for binding within the nucleosome borders, ∼40 bp away from the nucleosome start point (Fig. 6D). Other pioneering factors have also been shown to bind near the borders of the nucleosome, for instance, FOX factors which bind ∼60 bp from the center of the nucleosome, displacing linker histones and destabilizing the central nucleosome (Iwafuchi-Doi et al. 2016; Grossman et al. 2018). In contrast, when centering the MEL regions based on the TFAP2A motif, we did not observe a strong preference in the location of the nucleosome start point relative to the TFAP2A binding site (Fig. 6D), but in fact TFAP2A consistently binds in a wide range on and around the nucleosome middle point (Fig. 6E). Stabilizers, such as NFIB, have been reported to directly compete with the central nucleosomes to stabilize the accessible chromatin configuration (Denny et al. 2016; Grossman et al. 2018). Centering based on the SOX10 or TFAP2A motif hit revealed protection of Tn5 cutting on important nucleotides of the dimer motif (Supplemental Fig. S7A,B). We did not observe strong positional preferences of MITF and RUNX motifs relative to the nucleosome start or middle point (Supplemental Fig. S7C,D).

Altogether these data suggest that SOX10 functions as a pioneer in the CoRC of MEL enhancers, leading to their accessibility by binding to the central nucleosome, near the nucleosome start point. Conversely, TFAP2A appears to act as stabilizer of SOX-dependent nucleosome-depleted regions by binding around the nucleosome middle point, possibly going in competition with the central nucleosome.

### DeepMEL predicts evolutionary changes in MEL enhancer accessibility and activity

**Figure 7.**
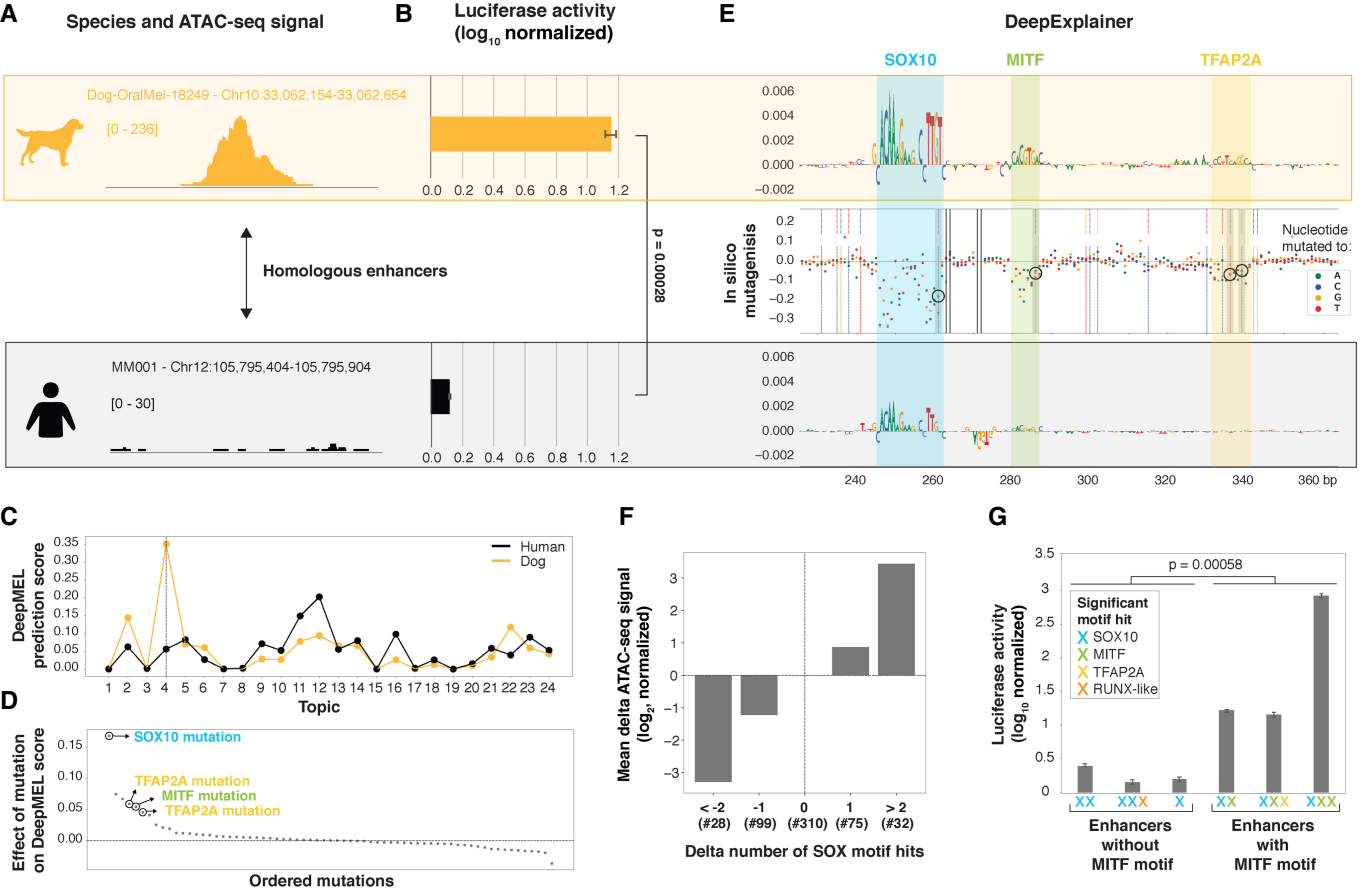
Predicting causal mutations of evolutionary changes in MEL enhancers. (*A*,*B*) Example region upstream of *APPL2* that is accessible (*A*) and active (*B*) in the MEL dog line Dog-OralMel-18249 but not in human MEL lines. (*C*) DeepMEL prediction score of each of the 24 topics for the dog and human *APPL2* enhancer. (*D*) Effect on topic 4 DeepMEL score on the dog sequence when in silico simulating each of the single detected point mutations between the dog and human *APPL2* enhancer. (*E*) DeepExplainer plots of the middle 120 bp of the dog and human *APPL2* enhancer. In the *middle*, the effect of each possible point mutation between the dog and human sequence on the MEL DeepMEL score was in silico calculated and is represented by colored dots depending on the nucleotide to which the original dog nucleotide was in silico mutated. Truly existing point mutations between the dog and human sequence are highlighted by color-coded vertical dashed lines. Four mutations that decrease the motif score of the SOX10, MITF, and TFAP2A motifs are highlighted by a gray box and are encircled. (*F*) Bar plot showing the mean effect on the log_2_ delta ATAC-seq signal of a non-human region compared to the human homolog depending on the number of SOX10 motif hits lost or gained. Only regions having no change in the number of significant TFAP2A, MITF, and RUNX motifs hits were used. The *y*-axis is normalized to the category with no changes in the number of significant SOX10 motif hits. The number of regions in each of the categories is mentioned (#). (*G*) Luciferase assay on six human or dog enhancers. Significant motif hits per enhancer are shown with colored crosses. For the luciferase assays: luciferase activity in MM001 is shown relative to *Renilla* signal and is log_10_ transformed. *P*-values were determined using Student's *t*-test, and the error bars represent the standard deviation over three biological replicates.

To further validate our findings on the MEL enhancer logic, we compared motif architectures between species and investigated how turnover of TF binding sites affects enhancer accessibility and function. To this end, we compared pairs of highly probable orthologous MEL enhancers that are only accessible in one of the species (Methods; Supplemental Fig. S8A). For example, an enhancer upstream of *APPL2* is predicted as a MEL enhancer in the dog line Dog-OralMel-18249 (topic 4 DL score of 0.35), whereas the orthologous enhancer in human is not accessible (Fig. 7A). Not only the accessibility of the human homolog was lost, but also its activity, as we confirmed by a luciferase assay (Fig. 7B). The topic 4 DeepMEL score for this enhancer was six times lower in human compared to dog (0.06 in human versus 0.35 in dog) (Fig. 7C), falling below the topic 4 significance threshold of 0.16, indicating that the model detected critical changes in the human enhancer sequence that could explain the loss of accessibility and activity of this MEL enhancer. The functional dog enhancer contains a SOX10, MITF, and TFAP2A binding site, which are all affected by substitutions in the nonfunctional human homologous sequence and might therefore be causal for the loss in accessibility (and activity) (Fig. 7D,E). The SOX10 motif mutation had the strongest effect, as it caused a 45% drop in the MEL-prediction score (Fig. 7D).

Next, we performed this analysis on a larger scale. First, per species pair, we observed that differences in DeepMEL predictions between species (delta-DeepMEL score) are highly predictive for differences in accessibility (Spearman's correlation of 0.43) (Supplemental Fig. S8B,C). Among the four studied regulators, mostly the disruption or gain of one or more SOX10 binding sites between orthologous enhancers quantitatively altered the ATAC-seq signal in a concordant way (Fig. 7F; Supplemental Fig. S8D), indicating that SOX10 mutations are most causal for changes in MEL enhancer accessibility, and possibly also in enhancer activity, as was the case in the *APPL2* enhancer above. However, concordance between accessibility and activity was not always observed (Supplemental Fig. S9). Furthermore, luciferase assays of six human or dog MEL-predicted enhancers suggested that enhancers with at least one MITF motif (*n* = 3) are significantly more active compared to enhancers without any MITF motif (*n* = 3) (Fig. 7G). Although the number of tested enhancers is small, this trend, together with the fact that MEL enhancers containing a MITF binding site showed increased H3K27ac signal (Supplemental Fig. S6C), indicates that MITF could function as an activator in MEL enhancers. Indeed, MITF has been shown to activate genes involved in pigmentation by recruitment of cofactors and chromatin remodeling complexes (Kawakami and Fisher 2017) and was previously classified as a TF involved in cofactor recruitment and activation (Grossman et al. 2018). SOX10 binding is insufficient but appears necessary for enhancer activity, because mutations in SOX10 binding sites disrupt enhancer activity in the *IRF4* case study (Fig. 3G).

In conclusion, DeepMEL provides a suitable platform to study the effect of evolutionary mutations on MEL enhancer accessibility and, in some cases, activity across species. Together, these results validate that SOX10 is crucial for enhancer accessibility in MEL enhancers, and necessary but insufficient for MEL enhancer activity, because activity appears to be mainly dependent on MITF binding.

## Discussion

Here, we present an in-depth study of melanoma enhancer logic, especially in enhancers specific to the melanocytic (MEL) state, by exploiting both cross-species data and machine learning. Although the MEL and MES melanoma cell states have been studied extensively on a transcriptomic and epigenomic level, the combinatorial code of binding sites of their regulatory factors in state-specific enhancers had not yet been explored. Understanding the enhancer logic and the mechanism by which TFs bind and direct active enhancers will become increasingly important, because it will be essential for the development of new therapies that influence cell state-specific enhancer functions in a targeted way (e.g., for enhancer therapy) (Johnson et al. 2008; Hamdan and Johnsen 2019), or to prioritize noncoding variants in whole-genome sequencing studies of personal or cancer genomes (Atak et al. 2019).

Predicting enhancers and determining their functional role within gene regulatory networks has been an active field for years. Despite the well-established power of cross-species approaches in this field, to our knowledge, a large comparative epigenomics study in melanoma has not yet been conducted, although several non-human models are commonly used in melanoma research (van der Weyden et al. 2016) and have been studied on an intra-species level (Rambow et al. 2008; Rosengren Pielberg et al. 2008; Sundström et al. 2012; Jiang et al. 2014; Seltenhammer et al. 2014; Kaufman et al. 2016; Hitte et al. 2019) or in relation to human melanoma (Egidy et al. 2008; Segaoula et al. 2018; Rahman et al. 2019). Here, we show that the MEL and MES states are conserved across species, as well as the key regulators of these states.

Despite their proven advantages, sequence-based comparative approaches have limited power to identify orthologous regulatory regions in distant species, in part because of the rapid evolution of distal enhancers (Dermitzakis and Clark 2002; Lindblad-Toh et al. 2011). Methods, such as enhancer element locator (EEL), try to tackle this question by aligning TF binding sites to identify conserved enhancer elements (Hallikas et al. 2006) or by calculating the co-occurrence of sequence patterns (Arunachalam et al. 2010). However, these methods are either supervised because they require user-provided PWMs (Hallikas et al. 2006), or it is difficult to extract the important biologically relevant features from these methods (Arunachalam et al. 2010). In addition, the identification and exact localization of important (de novo) TF binding sites within enhancers is complex because motif discovery tools are often dependent on user-provided databases and motif-specific thresholds. Recently, deep learning approaches, which are commonly used in disciplines such as speech recognition and image analysis, found their way into the regulatory genomics field to overcome these concerns (Park and Kellis 2015). Deep learning models, such as DeepBind, are particularly powerful in learning complex patterns by leveraging large epigenomics data sets; therefore, they are well suited to function as de novo motif detectors, as well as to uncover more complex sequence features (Alipanahi et al. 2015; Park and Kellis 2015). By designing DeepMEL, a multiclass, multilabel neural network trained on melanoma human regulatory topics of coaccessible regions, and by using the model interpretation tool DeepExplainer and our newly developed motif scoring scheme (Lundberg and Lee 2017; Lundberg et al. 2020), we were able to perform a thorough and unsupervised analysis of important TF binding sites in melanoma enhancers. Specifically, in MEL enhancers, our data suggest conserved cobinding of a CoRC of three main TFs, consisting of SOX10, TFAP2A, and MITF. DeepMEL also finds motifs for RUNX factors, but their role in the melanocyte or melanoma is less clear. Evidence for cobinding of SOX10, MITF, and TFAP2A was previously observed by enrichment of both MITF and TFAP2A motifs in SOX10 ChIP-seq data in melanoma cells (Laurette et al. 2015). We observed high flexibility in the organization of TF binding sites of the CoRC because eight different modalities were found, formed by all permutations of the CoRC factors, with the exception that all MEL enhancers contained at least one SOX10 binding site. MEL enhancers thereby adhere to a “mixed modes enhancer” model, a billboard-like model with mostly high flexibility in the TF motif organization, except for the ever-present SOX10 binding sites (Long et al. 2016). In addition, ChIP-seq data of MITF and TFAP2A indicated no indirect DNA binding of these CoRC factors within MEL enhancers, but that the bound TFs are largely determined by their individual motif presence. Although DeepMEL was trained on melanoma ATAC-seq data, the human- and pig-predicted MEL enhancers were also accessible in human and pig melanocytes, respectively, indicating that we could extend these observations on the MEL enhancer logic to enhancers in melanocytes, and that our methodology could be applied to nondisease states.

It is well established that distinct functional classes of TFs exist, with respect to enhancer binding. Pioneer TFs, such as POU5F1, SOX2, Grh-like TFs, and FOXA1, are able to bind nucleosomal DNA, leading to displacement of the nucleosome and facilitating the binding of other TFs to the accessible enhancer (Zaret and Carroll 2011; Long et al. 2016; Jacobs et al. 2018). SOX2 and other SOX factors have a HMG domain that interacts with the minor groove of the DNA, causing the DNA to bend in a 60°–70° angle, a property that has been suggested to contribute to the pioneering activity of SOX2, and possibly of other SOXs (Hou et al. 2017). Dodonova et al. (2020) indicate that SOX2 and SOX11 can bind to their binding motif on nucleosomal DNA and that they use their binding energy to initiate chromatin opening. However, there is still some dispute on the pioneering properties of SOX TFs, as another study classified SOXs as “migrant TFs,” that is, nonpioneering TFs that only bind sporadically to (non)-chromatinized DNA (Sherwood et al. 2014). Nonetheless, we find strong evidence for a pioneering function of SOX10 in MEL melanoma cells. Our current and previous study (Bravo González-Blas et al. 2019) have shown that knockdown of SOX10 induces closure of SOX10-bound ATAC-seq peaks containing a SOX10 motif. In fact, DeepMEL predicts SOX10 binding sites as essential for MEL enhancer accessibility. Next to pioneer factors, other functional classes of TFs exist, including factors that stabilize the accessibility of the nucleosome-depleted regions. TFAP2A was previously classified as such a chromatin stabilizer (Grossman et al. 2018), and it has been shown that evolutionary divergence from the TFAP2A consensus motif correlates with loss of chromatin accessibility and H3K27ac ChIP-seq signal (Prescott et al. 2015). These reports support our observations of TFAP2A as a stabilizer of SOX10-dependent accessible MEL enhancers, likely caused by direct competition of TFAP2A with the nucleosome, because TFAP2A binding sites were highly enriched at the predicted center of the central nucleosome. The dependence of SOX10 for opening MEL enhancers before TFAP2A binding is in line with the reported classification of TFAP2A as a “settler,” a TF whose binding depends predominantly on the accessibility of the chromatin at their binding sites (Sherwood et al. 2014).

Besides classifying accessible (orthologous) regions and predicting important TF motifs within them, DeepMEL is an accurate predictor of the effect of mutations on enhancer accessibility and, for some enhancers, also the activity. This was for instance the case for the *IRF4* MEL enhancer, where DeepMEL outperformed existing methods tested in Kircher et al. (2019). However, the other models in the benchmark were trained to predict the activity of a total of 20 regulatory regions ranging across different cell types, whereas our DL model is specialized for melanoma regulatory regions. This shows the value of using case-specific training data, such as the data set generated in this study for melanoma. Not all predicted MEL enhancers were in fact active, as MITF binding seems to be required to activate SOX10-dependent melanoma enhancers. Fufa et al. (2015) support this hypothesis, because activating SOX10-regions in mouse melanocytes showed significant enrichment of E-box motifs (bound by the bHLH protein family, which includes MITF), indicating that MITF cooperates with SOX10 to execute melanocyte-specific gene activation. In addition, MITF was previously classified as a TF involved in cofactor recruitment and activation (Kawakami and Fisher 2017; Grossman et al. 2018). Although SOX10 binding is not sufficient for enhancer activity, it appears to be necessary, because disruption of the SOX10 binding site in the *IRF4* enhancer had a strong effect on activity, probably owing to the reappearance of the central nucleosome.

In conclusion, the combination of comparative epigenomics with deep learning allowed us to perform an in-depth analysis of the melanoma enhancer logic. This work presents an overall framework that can be applied to decipher the enhancer logic in a cell type or cell state of interest, starting from the generation of an extensive cell type–specific (cross-species) epigenomics data set, all the way through the training and exploitation of a deep neural network to decode enhancer features across species, and to utilize it to assess the impact of *cis*-regulatory variation.

## Methods

### Cell culture

#### Human melanoma cell lines

Human melanoma cultures (MM lines) are short-term cultures derived from patient biopsies (Gembarska et al. 2012; Verfaillie et al. 2015). Cells were cultured at 37°C with 5% CO_2_ and were maintained in Ham's F10 nutrient mix (Thermo Fisher Scientific) supplemented with 10% fetal bovine serum (FBS; Thermo Fisher Scientific) and 100 µg mL^−1^ penicillin/streptomycin (Thermo Fisher Scientific).

#### Zebrafish melanoma cell lines

Experiments were performed as previously outlined (Ceol et al. 2011). Briefly, 25 pg of MCR:EGFP were microinjected together with 25 pg of Tol2 transposase mRNA into one-cell Tg(BRAFV600E); tp53^−/−^; mitf^−/−^ zebrafish embryos. Embryos were scored for melanocyte rescue at 48–72 h post-fertilization, and equal numbers were raised to adulthood (15–20 zebrafish per tank) and scored weekly (from 8 to 12 wk post-fertilization) or biweekly (>12 wk post-fertilization) for the emergence of raised melanoma lesions (van Rooijen et al. 2017). For in vitro culture, large tumors were isolated from MCR/MCR:EGFP (14–28 wk post-fertilization). Zebrafish were maintained under IACUC-approved conditions. Zebrafish primary melanoma ZMEL1 cell line was previously described (White et al. 2008, 2011), and EGFP 121-1, EGFP 121-2, EGFP 121-3, and EGFP 121-5, were generated as described (Heilmann et al. 2015; Wojciechowska et al. 2016). All cell lines were cultured in DMEM medium (Thermo Fisher Scientific) supplemented with 10% heat-inactivated FBS (Atlanta Biologicals), 1× GlutaMAX (Thermo Fisher Scientific), and 1% penicillin/streptomycin (Thermo Fisher Scientific), at 28°C, 5% CO_2_. Zebrafish melanoma lines were authenticated by qPCR and Western for EGFP transgene expression, and periodically checked for mycoplasma using the Universal Mycoplasma Detection Kit (ATCC).

#### Horse melanoma cell lines

The horse cell lines HoMel-L1 and HoMel-A1 are melanoma cell lines derived from a Lipizzaner stallion and Shagya-Arabian mare, respectively, and were established in Seltenhammer et al. (2014). Cells were cultured at 37°C with 5% CO_2_ in Roswell Park Memorial Institute (RPMI) medium (Thermo Fisher Scientific) supplemented with 10% FBS (Thermo Fisher Scientific) and 1% penicillin/streptomycin (Thermo Fisher Scientific).

#### Pig melanoma and melanocyte cell line

The immortal line of pigmented melanocytes (PigMel) was previously derived (Julé et al. 2003), and the 30-d-old piglet primary melanoma cells (MeLiM) were isolated as described (Egidy et al. 2008). PigMel cells were cultured at 37°C with 10% CO_2_ in MEM medium supplemented with 1× MEM nonessential amino acids (Thermo Fisher Scientific), 1 mM Na pyruvate, 2 mM glutamine, 100 units/mL penicilin/streptomycin (Thermo Fisher Scientific), 10% FCS and 3.7 g/mL Na bicarbonate. MeLiM cells were cultured in DMEM high glucose (Thermo Fisher Scientific), 10% FCS, Pen/Strep, and 5% CO_2_.

#### Dog melanoma cell lines

The dog cell lines Dog-IrisMel-14205 and Dog-OralMel-18249 were established by Aline Primot, and were derived from an uveal melanoma from a beagle crossed dog and an oral melanoma from the palate of a Shih Tzu, respectively. Cells were cultured at 37°C with 5% CO_2_ in Ham's F-12 Nutrient Mixture medium (Thermo Fisher Scientific) supplemented with 10% FBS (Thermo Fisher Scientific) and 1% penicillin/streptomycin (Thermo Fisher Scientific).

#### Mouse melanoma cell lines

The mouse melanoma cell line was generated as described (Dankort et al. 2009). Cells were cultured at 37°C with 5% CO_2_ in Dulbecco's Modified Eagle Medium (DMEM) (Thermo Fisher Scientific) supplemented with 10% FBS (Thermo Fisher Scientific) and 1% penicillin/streptomycin (Thermo Fisher Scientific).

### Knockdown experiments

SOX10, TFAP2A, and the control knockdown (KD) were performed in MM001 using a SMARTpool of four siRNAs against, respectively, *SOX10* (SMARTpool: ON-TARGETplus SOX10 siRNA, number L017192-00-0005, Dharmacon), *TFAP2A* (SMARTpool: ON-TARGETplus TFAP2A siRNA, number L-006348-02-0005, Dharmacon), and a negative control pool (ON-TARGETplus nontargeting pool, number D-001810-10-05, Dharmacon) at a concentration of 20 nM for SOX10-KD, and 40 nM for TFAP2A-KD and the control using as medium Opti-MEM (Thermo Fisher Scientific) and omitting antibiotics. The cells were incubated for 72 h before processing.

### OmniATAC-seq data generation, data processing, and follow-up analyses

#### OmniATAC-seq on mammalian lines

Omni-assay for transposase-accessible chromatin using sequencing (OmniATAC-seq) was performed as described previously (Corces et al. 2017). After the final amplification was done with the additional number of cycles, samples were cleaned-up by MinElute and libraries were prepped using the KAPA Library Quantification Kit as previously described (Corces et al. 2017). Samples were sequenced on a HiSeq 4000 or NextSeq 500 High Output chip.

#### ATAC-seq on zebrafish lines

Fifty thousand cells per line were lysed and subjected to a tagmentation reaction and library construction as described in Buenrostro et al. (2013). Libraries were run on an Illumina HiSeq 2000.

#### Data processing of ATAC-seq and OmniATAC-seq samples

Paired-end or single-end reads were mapped to the human genome (hg19-GENCODE v18) using Bowtie 2 (v2.2.6) (Langmead and Salzberg 2012) or STAR (v2.5.1b) (Dobin et al. 2013) to species-specific genomes, which were downloaded from UCSC (https:// hgdownload.soe.ucsc.edu/downloads.html) (for human: hg19-GENCODE v18; for dog: canFam3; for horse: equCab2; for pig: susScr11; for mouse: mm10; for zebrafish: danRer10) and by applying the parameters ‐‐alignIntronMax 1 and ‐‐alignIntronMin 2. For the human data, we used hg19 as genome assembly instead of the more recent GRCh38 assembly because i-cisTarget (Herrmann et al. 2012; Janky et al. 2014; Imrichová et al. 2015) and GREAT (McLean et al. 2010) are or were not (yet) available for GRCh38 at the time of the analyses. However, the use of GRCh38 instead of hg19 would not significantly affect conclusions. We, for instance, validated this by rescoring MEL-predicted regions by DeepMEL in MM057 after liftOver (Kuhn et al. 2013) from hg19 to GRCh38, in which we observed that changing genome assembly yields the same DeepMEL score for all 4244 regions except for eight of them. Also note that for MM029, two biological replicates were used. Mapped reads were sorted using SAMtools (v1.8) (Li et al. 2009), and duplicates were removed using Picard MarkDuplicates (v1.134). Reads were filtered by removing mitochondrial reads and filtering for *Q* > 30 using SAMtools. BAM files of technical replicates of the same cell line were merged at this point using SAMtools merge. Peaks were called using MACS2 (v2.1.2) (Gaspar 2018) callpeak using the parameters -q 0.05, ‐‐nomodel, ‐‐call-summits, ‐‐shift -75 ‐‐keep-dup all and ‐‐extsize 150 per sample. Blacklisted regions (ENCODE) and peaks overlapping with alternative chromosomes and ChrM were removed. Summits were extended by 250 bp up- and downstream using slopBed (BEDTools; v2.28.0) (Quinlan and Hall 2010), providing human chromosome sizes. Peaks were normalized for the library size using a custom script, and overlapping peaks were filtered using the peak score by keeping the peak with the highest score. Normalized bigWigs were either made from normalized bedGraphs using as scaling parameter (-scale) 1 × 10^6^/(number of nonmitochondrial mapping reads); or made by bamCoverage (deepTools, v3.3.1) (Ramírez et al. 2016), using as parameters ‐‐normalizeUsing None, -bl EncodeBlackListedRegions ‐‐effectiveGenomeSize 2913022398 and as scaling parameter (-scaleFactor) 1/(RIP/1 × 10^6^), in which RIP stands for the number of reads in peaks.

#### HOMER on human and dog differential accessible peaks

Count matrices were produced by featureCounts (v1.6.5) (Liao et al. 2014) for five melanocytic (MEL) and five mesenchymal-like (MES) lines for human, and for Dog-OralMel-18249 and Dog-IrisMel-14205 for dog. Differential peaks were identified using DESeq2 (v1.22.2, R v3.5.2) (R Core Team 2018; Love et al. 2014) with a log_2_FC higher than 2.5 and a *P*_Adj_ lower than 0.0005. HOMER (Heinz et al. 2010) was performed on the differentially accessible regions using findMotifsGenome.pl, providing the differential regions as a BED file and a FASTA file of the human or dog genome, with parameters -mask, -size given, and -len 6,8,10,11,12,17,18.

#### Defining sets of alignable and conserved accessible ATAC-seq regions

ATAC-seq regions of non-human species were defined as alignable regions when they could be converted to hg19 coordinates using liftOver (Kent-tools, -minMatch = 0.1) (Kuhn et al. 2013) by providing the appropriate liftOver chain (UCSC). Alignable regions were intersected with accessible peaks in human using intersectBed (BEDTools, v2.28.0) (Quinlan and Hall 2010) with -f 0.6 to define sets of conserved accessible regions across species.

#### Clustering of species based on globally alignable ATAC-seq regions

Per species, a count matrix was made on the alignable union ATAC-seq regions by featureCounts (v1.6.5) (Liao et al. 2014). The count matrices of different species were merged and the final count matrix was CPM normalized (edgeR v3.22.5, R v3.5.2) (Robinson et al. 2010; R Core Team 2018), followed by quantile normalization. A principal component analysis (PCA) on the normalized count matrix was performed using irlba (v2.3.3, R v3.5.2) (Baglama and Reichel 2005).

### Branch length scoring across species

Conserved accessible ATAC-seq regions were identified as described above, and for each of the species, the set of conserved accessible regions was converted to the coordinate system per species and FASTA sequences were retrieved. All sequences were scored with the cisTarget motif collection (v8) (http://iregulon.aertslab.org/collections.html) (Herrmann et al. 2012; Janky et al. 2014; Imrichová et al. 2015) containing 20,003 TF position-weight matrices (PWMs) using Cluster-Buster (Frith et al. 2003) with parameters -m 0, -c 0, and -r 10000. For each motif, the highest *cis*-regulatory module (CRM) score per conserved accessible sequence was used to calculate the branch length score (BLS) across species according to Stark et al. (2007) and Jacobs et al. (2018). The branch length was taken from the phylogenetic data from http://hgdownload.cse.ucsc.edu/goldenpath/hg19/phyloP100way/ (UCSC). The sum of the BLSs for all the conserved accessible sequences across the mammalian or all six species was used as a total score for each motif. We normalized these scores by performing BLS on a shuffled variant of all sequences by shuffleseq (EMBOSS, v6.6.0.0), keeping the same base-pair compositions and sequence lengths, and subtracting the shuffled BLS from the true BLS per motif.

### CisTopic analysis to obtain sets of coaccessible regions in human OmniATAC-seq data

To apply cisTopic (Bravo González-Blas et al. 2019), a tool designed for single-cell ATAC-seq analysis, we first simulated single cells from the bulk OmniATAC-seq data of the 16 human melanoma lines via bootstrapping. Per cell line, 50 simulated single-cell BAM files were generated containing each 50,000 random reads that were bootstrapped from the bulk BAM files. These simulated single-cell BAM files were provided as input for cisTopic (v0.2.0, R v3.4.1) (R Core Team 2017), together with the merged BED file of ATAC-seq regions across all 16 samples, after removing blacklisted regions (ENCODE). We ran cisTopic (parameters: α = 50/T, β = 0.1, burn-in iterations = 500, recording iterations = 1000) for models with a number of topics (sets of coaccessible regions) between 2 and 30 (2 by 2). The best model, containing 24 topics, was selected on the basis of the highest log-likelihood. Topics were binarized using a probability threshold of 0.995 (resulting in a total of 35,940 binarized topic regions across the 24 topics), and we performed motif enrichment analysis with cisTarget (Imrichová et al. 2015).

### Deep learning

#### Data preparation

The deep learning (DL) model, DeepMEL, was trained on the binarized regions of the 24 topics obtained from the cisTopic analysis explained above. To increase the amount of training data, the 500-bp regions in the merged BED file of all 339,099 ATAC-seq regions across the 16 human cell lines (see “Data processing of ATAC-seq and OmniATAC-seq samples”), were augmented by extending them to 700 bp around the summit and sliding a 500-bp window over these elongated regions with a 10-bp stride. This augmented master region BED file was intersected with each topic BED file separately (using BEDTools) (Quinlan and Hall 2010), and a region was labeled with a topic number if there was at least 60% overlap. If regions overlapped with multiple topics, they were assigned with multiple topic labels, allowing for a multilabel and multiclass DL model. This augmentation and intersection resulted in 696,654 training regions in total, excluding the 58,086 regions on Chr 2 that were used for testing.

#### DeepMEL model architecture and training parameters

The DeepMEL architecture was built with four layers between input and output layer: a Conv1D layer (containing 128 filters and setting the parameters kernel_size as 20, the strides as 1 and the activation as relu), MaxPooling1D layer (with the pool_size 10 and strides 10), TimeDistributed Dense layer together with Bidirectional LSTM layer (with 128 unit and setting the dropout as 0.1 and the recurrent_dropout as 0.1), and Dense layer (with 256 units and setting the activation as relu). After MaxPooling1D, Bidirectional LSTM, and Dense layer, a Dropout layer was used each time with the fraction of dropout set as 0.2, 0.2, and 0.4, respectively. For each region in the training data, DeepMEL takes the one-hot encoded (500 bp × 4 nt) forward and reverse strand and passes them separately through the model. To make the final prediction, DeepMEL takes the average activation (*average* function) of the neurons in the final Dense layer (which contains 24 units corresponding to the 24 topics; with a sigmoid activation function). The model was compiled using the Adam optimizer with the default learning rate, which is 0.001. To calculate the loss, the binary cross entropy (binary_crossentropy) was used. The model was trained for two epochs with a batch size of 128, which took 67 min. Keras 2.2.4 (https://keras.io) with tensorflow 1.14.0 (Abadi et al. 2016) was used. A Tesla P100-SXM2-16GB GPU was used for training on VSC servers (Flemish Supercomputer Center).

#### Performance evaluation

The performance of the model was evaluated for each topic separately because it was a multilabel classifier. The auROC and auPR were calculated for the combined training and validation data (regions on all chromosomes except Chr 2), test (regions on Chr 2), and label-shuffled regions.

#### Converting convolution filters to PWMs, filter-topic assignment, and filter annotation

Filters of the convolution layer were converted to position-weight matrices (PWMs) by the following strategy: (1) 4,000,000 unique 20-bp-long (size of the filters) sequences were randomly generated; (2) the activation score of each filter for each sequence was calculated and the top 100 sequences were selected; (3) a count matrix was generated from these 100 sequences obtained for each filter; and (4) finally, the count matrices were converted into PWMs. To assign the filters to topics, a similar strategy that is mentioned in Basset (Kelley et al. 2016) was used. After setting the activation score of a filter to its mean activation score over all the sequences, the loss/accuracy score on the prediction was calculated for each topic. Filters were ordered based on their effect on a certain topic. To annotate the filters to known transcription factor binding motifs, the Tomtom motif annotation tool (Gupta et al. 2007) was used together with our curated cisTarget motif collection (v9) (http://iregulon.aertslab.org/collections.html) (Herrmann et al. 2012; Janky et al. 2014; Imrichová et al. 2015) of 24,453 PWMs (cutoff for the *Q*-value was set to 0.3).

#### DeepExplainer

From the 35,940 topic regions that were obtained after binarization of the 24 topics within the selected cisTopic model (see methods on cisTopic analysis above), 500 regions were randomly selected to initialize the DeepExplainer pipeline (Lundberg and Lee 2017). A hypothetical importance score for each position of the sequence of interest was calculated for any of the 24 topics. For each sequence, these DeepExplainer-obtained importance scores were multiplied by the one-hot encoded matrix of the sequences. Finally, the 500-bp sequences were visualized by adjusting the nucleotide heights based on their importance score by using the modified viz_sequence function from the DeepLift repository (Shrikumar et al. 2017).

#### In silico saturation mutagenesis

In silico saturation mutagenesis of a region was performed by separately changing each nucleotide on the 500-bp sequence into the three other nucleotides and scoring these mutated sequences with DeepMEL. The delta prediction score for each mutation was calculated for each of the 24 topics by comparing the prediction score of the mutated sequence relative to the prediction score for the initial sequence. For the *IRF4* enhancer case, the actual *IRF4* enhancer sequence used in the in vitro saturation mutagenesis assay (Chr 6: 396,143–396,593) overlapped with a predicted MEL enhancer in human MEL cell lines in our cohort (Chr 6: 396,135–396,636). The delta prediction score of topic 4 (MEL topic) was calculated following an in silico saturation mutagenesis on this region, and a Pearson's correlation was calculated on the overlapping nucleotides between the in silico and in vitro assays (451 bp).

#### Motif scoring method

We designed an optimized motif scoring method, in which activation scores of the filters on each sequence are multiplied by the DeepExplainer importance scores of the sequence. Then, after the output of this multiplication was normalized, a threshold was calculated for each motif by comparing MEL and MES enhancers. This approach yielded significant motif hits with their precise location.

#### Nucleosome positioning

Nucleosome start and middle point predictions were calculated by using the executable nucleosome prediction tool Kaplan_v3 (Kaplan et al. 2009) that takes just the DNA sequence and calculates the nucleosome positioning for each nucleotide. To get more precise results, as the authors of Kaplan_v3 suggest, enhancers were extended 3 kb from both ends. After obtaining the predictions, the middle 500-bp part of the 6.5-kb nucleosome prediction score was used.

#### Tn5 footprinting

Footprints of the Tn5 were determined by inferring Tn5 cut sites from the start point of each ATAC-seq read in a BAM file using a custom script.

### AUROC on human and dog of DeepMEL and Cluster-Buster

The performance of DeepMEL to discriminate between MEL and MES regions in human and dog was calculated by scoring the top 5000 differential MEL and MES regions in human and dog (described above) with DeepMEL and calculating the precision of correct assignment (i.e., topic 4 score for the MEL regions and topic 7 scores for the MES regions). The performance of DeepMEL was compared with the motif scoring tool Cluster-Buster (Frith et al. 2003) by scoring the same sets of regions with Cluster-Buster using a merged motif file of (some of) the top filters identified by the model in either topic 4 or topic 7. The obtained CRM scores were used to estimate the performance of Cluster-Buster.

### Identification of homologous MEL genes and MEL enhancers

To identify genes differentially expressed in human MEL cell lines, we performed DESeq2 (v1.22.2, R v3.5.2) (R Core Team 2018; Love et al. 2014) on RNA-seq data of seven MEL (MM031, MM034, MM057, MM074, MM087, MM118, MM164) and five MES (MM029, MM099, MM116, MM163, MM165) human lines. Three hundred seventy-nine genes were found differentially expressed in MEL lines (log_2_FC > 2.5 and *P*_Adj_ < 0.005). We converted the gene symbols to Ensembl gene IDs using biomaRt (v2.38.0, R v3.5.2) (Durinck et al. 2005) and found back the genomic locations of the genes using GenomicFeatures (v1.34.8, R v3.5.2) (Lawrence et al. 2013). For the human differential MEL genes with at least one MEL-predicted peak in their extended gene locus (200 kbp upstream and downstream), the homologous genes in the other six species were identified using biomaRt to convert the human Ensembl gene IDs to Ensembl gene IDs of the other species. We identified the MEL enhancers that overlapped with the extended gene loci of each of the homologous genes using BEDTools intersect (Quinlan and Hall 2010). liftOver (-minMatch = 0.1) (Kuhn et al. 2013) was used to calculate the number of these regions that could be identified by performing coordinate conversion.

### Correlation of MEL enhancers using deep layers of DeepMEL

Conserved accessible MEL enhancers in the extended loci of conserved MEL-specific genes across the six species (see above) were scored by DeepMEL. A matrix was generated consisting of a score for each of the 256 nodes in the Dense layer for each of the regions. A Pearson's correlation matrix was generated to calculate the pairwise similarity between each of the regions.

### Genome-wide prediction of MEL enhancers

The first chromosome of the human genome (hg19) was tiled with a sliding window of 500 bp and a 100-bp shift using BEDTools makewindows (v2.28.0) (Quinlan and Hall 2010). Tiles containing “N” were deleted and the remaining tiles were scored by DeepMEL, and the number of MEL-predicted tiles (topic 4 score > 0.16) was calculated.

### Mutations in orthologous enhancers across species

We defined highly probable orthologous MEL enhancers between human and another species as regions that were predicted as MEL in one species and for which there was a stringent liftOver (-minMatch = 0.995) (Kuhn et al. 2013) and high sequence identity, that is, >80% after pairwise alignment via needle (EMBOSS, v6.6.0.0) (Madeira et al. 2019), using parameters -gapopen 10.0 -gapextend 0.5, in the other species. featureCounts (v1.6.5) (Liao et al. 2014) was used to generate count matrices per species on these regions, which was followed by library size normalization. Delta ATAC-seq scores were calculated for the pairs of orthologous regions by dividing the normalized counts of the two species (human counts/non-human counts) after adding a pseudocount. Mutations were identified by alignment via needle, using the parameters -gapopen 10.0 and -gapextend 0.5.

### Luciferase assay

Six MEL-predicted enhancers (three in the dog line Dog-OralMel-18249 and three in the human line MM001) were synthetically generated and cloned into a pTwist ENTR plasmid (Twist Bioscience) via Twist Bioscience. Regions were transferred from the Gateway entry clone into the destination vector (pGL4.23-GW, Addgene) via a LR reaction by mixing 2 µL of the entry clone (100 ng/µL) with 1 µL of the destination plasmid (150 ng/µL), 1 µL TE buffer, and 1 µL LR enzyme (LR Clonase II Plus enzyme mix, Thermo Fisher Scientific), and incubating this mixture at 25°C. Afterwards, 1 µL of Proteinase K (Thermo Fisher Scientific) was added and reactions were incubated for 1 h at 37°C for 10 min. Then, 3 µL of each LR reaction was transformed into 50 µL of Stellar competent cells (Takara Bio) via heat shock. Next, 200 µL of SOC medium was added and the cells were incubated for 1 h in a shake incubator at 37°C, before plating the transformed cells on LB agar plates with 1/1000 carbenicillin and incubation overnight at 37°C. The next day, one colony per construct was picked and grown overnight in 5 mL of LB medium with 1/1000 carbenicillin in a shake incubator at 37°C before plasmid extraction using the NucleoSpin Plasmid Transfection-grade kit (Macherey-Nagel). For each construct, three biological replicates were performed by transfecting the plasmids into 80% confluent cells of MM001 in a 24-well plate. Per transfection, 400 ng of the construct was transfected together with 40 ng of *Renilla* plasmid (Promega) using lipofectamine 2000 (Thermo Fisher Scientific). Luciferase activity of each construct was measured using the Dual-Luciferase Reporter Assay (Promega) according to the manufacturer's instructions. Enhancer luciferase activity was normalized against the *Renilla* luciferase activity.

### Publicly available data used in this work

SOX10 ChIP-seq and MITF ChIP-seq data on the 501Mel melanoma cell lines were downloaded as raw FASTQ files from the NCBI Gene Expression Omnibus (GEO; https://www.ncbi.nlm.nih.gov/geo/) through accession number GSE61965 (Laurette et al. 2015) and were mapped to the human genome using Bowtie 2 (v2.1.0) (Langmead and Salzberg 2012) and peaks were called by MACS2 (v2.1.1) (Gaspar 2018). TFAP2A ChIP-seq data on human primary melanocytes from neonatal foreskin were retrieved from Seberg et al. (2017) (GSE67555) as a BED file, which was converted to a bedGraph and bigWig using the peak height from the BED file. Histone H3 at lysine 27 (H3K27ac) and H3 monomethylation at K3 (H3K4me1) ChIP-seq data for MM001 (GSE60666); and RNA-seq data (for MM031, MM034, MM057, MM074, MM087, MM099, and MM118 downloaded from GSE60666; for MM029, MM116, MM0163, MM164, and MM165 from GSE134432) were processed as explained in Verfaillie et al. (2015). OmniATAC-seq data for the human lines MM001, MM011, MM029, MM031, MM074, MM057, MM087, and MM099 were obtained through GSE134432 (Wouters et al. 2020) and were processed as described above in “Data processing of ATAC-seq and OmniATAC-seq samples”; which was also the case for ATAC-seq data from normal human melanocytes on foreskin (NHM1), which were downloaded as raw FASTQ files from GSE94488 (GSM2476338) (Fontanals-Cirera et al. 2017). The massively parallel reporter assay (MPRA) data on the *IRF4* enhancer was downloaded from https://mpra.gs.washington.edu/satMutMPRA/ and was processed as described above.

## Data access

All raw and processed sequencing data generated in this study have been submitted to the NCBI Gene Expression Omnibus (GEO; https://www.ncbi.nlm.nih.gov/geo/) under accession number GSE142238. This includes OmniATAC-seq data of human melanoma cell lines (MM029, MM034, MM052, MM116, MM118, MM122, MM163, MM164, MM165; data for the other lines used in this study were published before [see “Publicly available data used in this work”]), two dog melanoma cell lines, two horse melanoma cell lines, one pig melanoma sample, one pig melanocyte cell line, and one mouse melanoma cell line; ATAC-seq data of four zebrafish cell lines; and OmniATAC-seq data of SOX10 and TFAP2A knockdown in the human melanoma cell line MM001. The DeepMEL model was deposited in Kipoi (Avsec et al. 2019) (http://kipoi.org/models/DeepMEL/). Code and custom scripts for training DeepMEL, DeepMEL predictions, DeepExplainer usage, and BLS scoring are provided in GitHub (https://github.com/aertslab/DeepMEL) and as Supplemental Code.

## Competing interest statement

The authors declare no competing interests.

## Supplementary Material

Supplemental Material
