# Supplementary material for: Cross-species analysis of enhancer logic using deep learning
Source: Genome Res. 2020 Dec;30(12):1815–34. doi: 10.1101/gr.260844.120 (PMC7706731; doi:10.1101/gr.260844.120)
Supplement: Supplemental Material [file supp_30_12_1815__index.html]

Cross-species analysis of enhancer logic using deep learning — Cross-species analysis of enhancer logic using deep learning — Supplemental Material 

# Cross-species analysis of enhancer logic using deep learning

## Supplemental Material

- Supplemental\_Figures.docx
- Supplemental\_Note.docx
- Supplemental\_Table\_S1.xlsx
- Supplemental\_Code.zip
