# Supplementary material for: Cross-species analysis of enhancer logic using deep learning
Source: Genome Res. 2020 Dec;30(12):1815–34. doi: 10.1101/gr.260844.120 (PMC7706731; doi:10.1101/gr.260844.120)
Supplement: Supplemental Material [file supp_gr.260844.120_Supplemental_Figures.docx]

## **Supplementary figures**

##



**Figure S1.** Clustering of melanoma samples across species. (*A*) Distribution of the number of ATAC-seq peaks called per sample across species. (*B*) Scatterplot of the number of ATAC-seq peaks versus the genome size, where each dot represents a non-human sample. A linear trendline is plotted and the R-squared value is indicated. (*C*) Scatterplot of the number of ATAC-seq peaks versus the fraction of reads in peaks (FRiP), where each dot represents a human sample. A linear trendline is plotted and the R-squared value is indicated. (*D*) PCA plot of the human melanoma lines using normalised ATAC-seq read counts on the 50,000 most variable peaks. (*E*) Expression of MEL and MES signature genes (Verfaillie et al., 2015) on 12 of the human cell lines. (*F*) 339,099 human ATAC-seq regions coloured based on whether they are not alignable, alignable or conserved accessible in each of the other non-human species. (*G*) Number of human ATAC-seq regions that are conserved accessible in 0 (i.e. human-specific) to 5 different species. ChIPseeker results are shown for the 10,592 human regions that are conserved accessible across all mammalian species. (*H*) ATAC-seq and H3K27ac ChIP-seq signal of the human line MM001 on the 10,592 conserved accessible regions and on all human regions. (*I*) ATAC-seq signal of mouse, pig, horse (HoMel-L1) and dog (Dog-OralMel-18249) melanoma samples on the 10,592 conserved accessible regions and on all regions of the specific species. (*J*) Boxplots of the phasCons and phyloP score of the set of regions that are alignable or conserved accessible across the five mammalian species or across all the six studied species. (*K*) PCA plot of the mammalian melanoma lines based on the 303,392 alignable regions. (*L*) PCA plot of the mammalian melanoma lines based on the 10,592 conserved regions. (*M*) PCA plot of all cross-species melanoma lines based on the 116 globally conserved accessible regions. (*N*) Histogram of the cumulative BLS for 20,003 motifs on 5,196 conserved accessible non-promoter regions (i.e. no proximal promoters situated < 1 kbp from TSS) in the MEL lines across the mammalian species. The first hit of recurrent TF binding motifs within the top 4% conserved motifs is indicated as a cross and is accompanied by the logo of the motif.


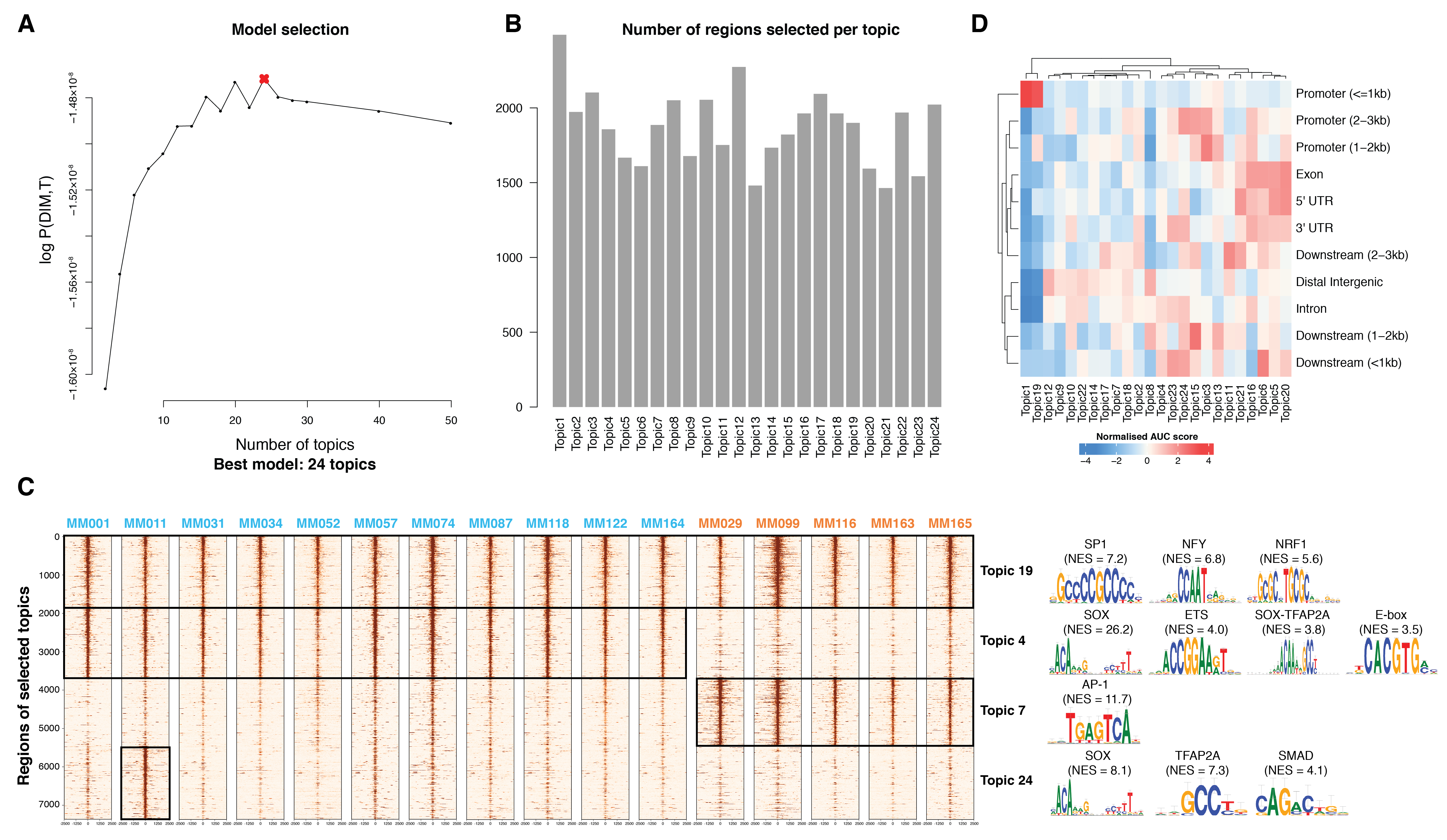


**Figure S2.** Topic modeling on ATAC-seq data of 16 human melanoma cell lines. (*A*) cisTopic model with 24 topics was selected based on the log-likelihood in the last iteration. (*B*) Number of regions per topic after topic binarisation. (*C*) Coverage heatmaps of ATAC-seq data on regions of four selected topics: a promoter topic (topic 19), the MEL-specific topic (topic 4), the MES-specific topic (topic 7) and a cell line-specific topic (topic 24, specific to MM001). Key motifs found enriched in the selected topics by i-cisTarget, accompanied by their NES score. (*D*) Topic annotation heatmap.





**Figure S3.** Hyperparameter optimisation of DeepMEL. (*A-D*) auROC and auPR for topic 1, topic 4 and topic 7 prediction on train, validation, test and shuffled data when varying the number of filters (*A*), the filter size (*B*), the length of the input DNA sequences (*C*), the model architecture (*D*). (*E*) auROC and auPR of the final model (DeepMEL) before and after adding validation data to the training set.


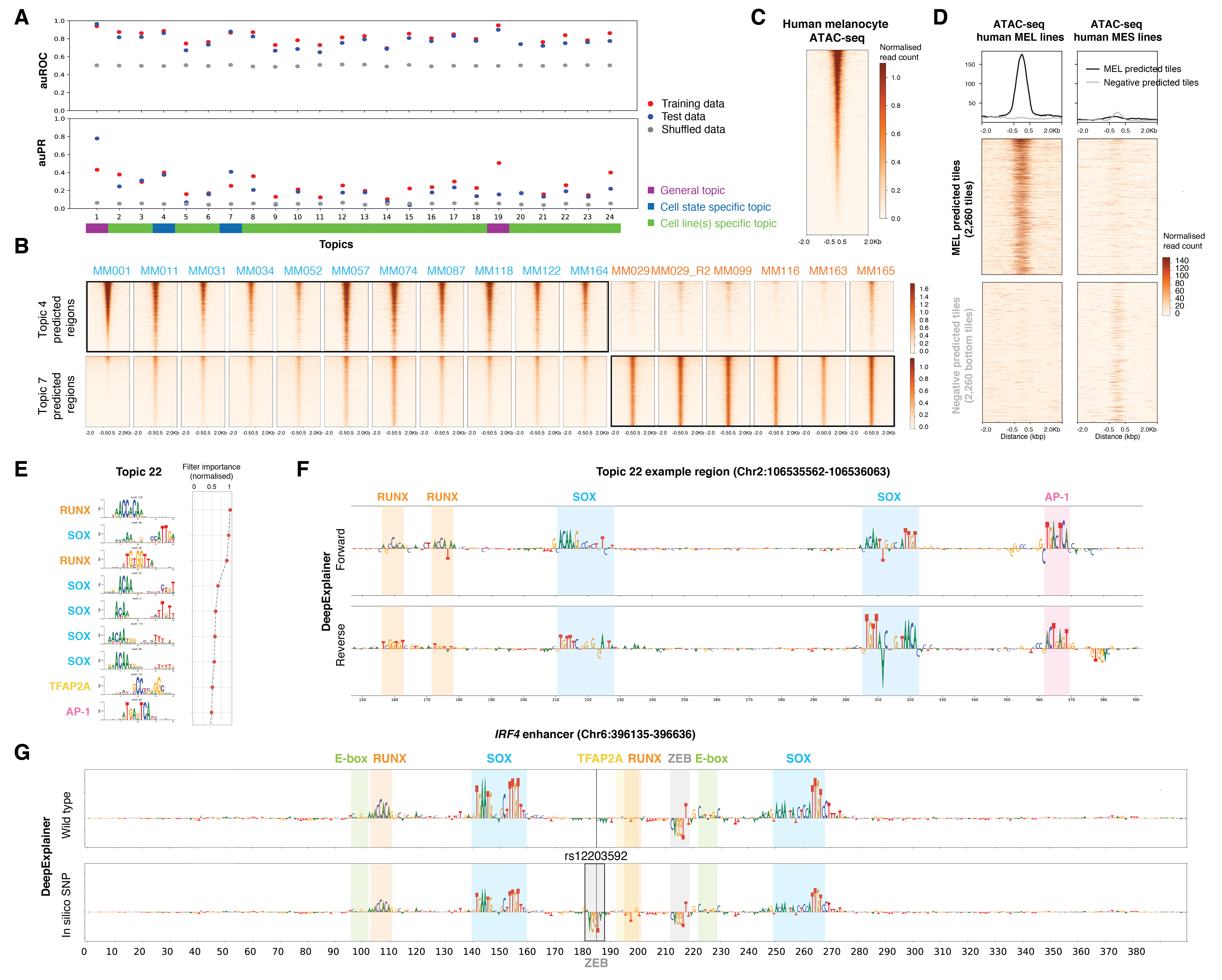


**Figure S4.** DeepMEL reveals important features in melanoma regulatory classes. (*A*) auROC and auPR curve of DeepMEL for training, test and shuffled data for classifying regions of the 24 topics. (*B*) Coverage heatmaps of ATAC-seq data of the 16 human lines on regions of the MEL-specific topic (topic 4) and MES-specific topic (topic 7). (*C*) ATAC-seq signal of human melanocytes on topic 4 predicted regions by DeepMEL in MM001, ordered according to the melanocyte ATAC-seq signal. (D) Genome-wide scoring of may be useful for species where no ATAC-seq data of melanoma is available. Such a scoring yields high recall (86%, proportion of MEL-predicted ATAC-seq regions being found chromosome-wide) and precision (69%, proportion of MEL-predicted chromosome-wide tiles that were also accessible in human MEL lines) when applying DeepMEL to 500 bp sliding windows on Chromosome 1 in human. To visualise this, ATAC-seq signal of merged human MEL lines and merged human MES lines is shown on 2,260 MEL-predicted tiles (DeepMEL score of topic 4 > 0.16) when scoring 500 bp sequences tiled across Chromosome 1 in the human genome by DeepMEL, and on the 2,260 tiles with the lowest topic 4 prediction score. Heatmaps are coloured by normalised read counts and aggregation plots are shown on top. (*E*) The top features learned by DeepMEL to classify topic 22 regions (regions specific to the intermediate state). (*F*) DeepExplainer plots on the forward and reverse strand of an example topic 22 region. The enhancer contains both AP-1 and SOX motifs. (G) Enhancer accessibility and activity on the *IRF4* enhancer were also influenced by mutations that create a repressor binding motif. This was the case for a SNP associated with freckles, brown hair and high sensitivity of the skin to sun exposure (rs12203592, SNPedia), creating a ZEB/SNAI-like motif (CAGGT) that negatively contributes to the MEL topic score of this enhancer. Indeed, ZEB factors have been reported to act as transcriptional repressors by interaction with the corepressor CtBP (Postigo and Dean 1999), and mutations in the binding motif of the transcriptional repressor SNAI2 have been shown to increase chromatin accessibility (Prescott et al. 2015). DeepExplainer profiles of wildtype *IRF4* enhancer and after in silico introduction of the rs12203592 SNP, which creates a ZEB-like motif.


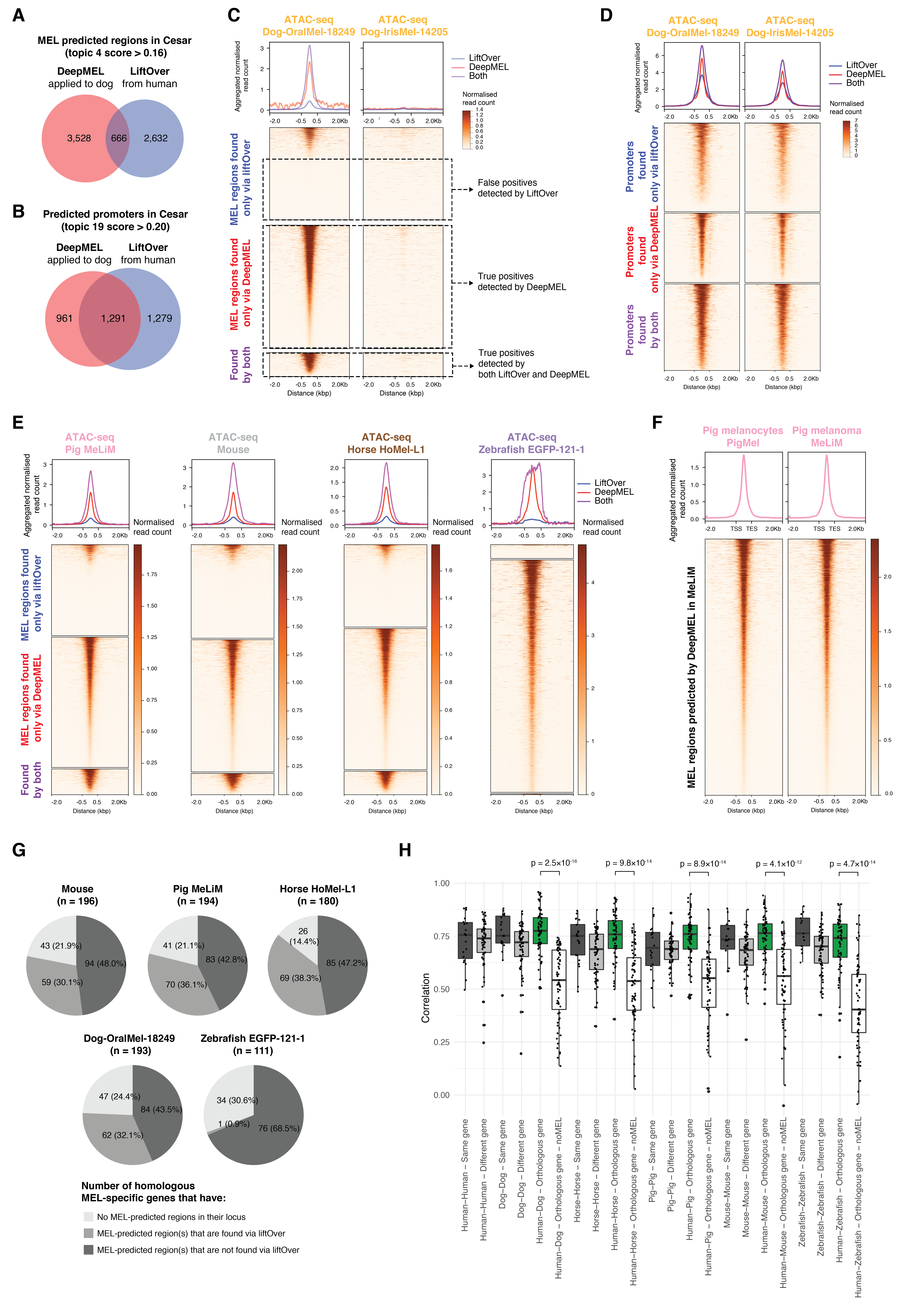


**Figure S5.** Genome-wide MEL enhancer predictions using DeepMEL. (*A, B*) (*A*) Venn diagram of the number of topic 4 (MEL-specific) and (*B*) topic 19 (promoter) regions predicted by DeepMEL in the dog line ‘Dog-OralMel-18249’ and of dog regions found by liftOver of the human MEL regions. (*C, D*), Heatmaps of ATAC-seq signal of the dog lines ‘Dog-OralMel-18249’ and ‘Dog-IrisMel-14205’ on (*C*) MEL-predicted and (*D*) promoter-predicted regions found via liftOver (blue), MEL regions predicted by DeepMEL (red) and MEL regions identified by both methods (purple). Heatmaps are coloured by normalised read counts and ordered according to the ATAC-seq signal in ‘Dog-OralMel-18249’. Aggregation plots are shown on top. (*E*) Heatmaps of ATAC-seq signal of pig, mouse, horse and zebrafish on MEL regions regions found via liftOver (blue), MEL regions predicted by DeepMEL (red) and MEL regions identified by both methods (purple). Heatmaps are coloured by normalised read counts and ordered according to the ATAC-seq signal and aggregation plots of are shown on top. (*F*) ATAC-seq signal of pig melanocytes (‘PigMel’) and pig melanoma (‘MeLiM’) on MEL regions predicted in MeLiM. Heatmaps are coloured by normalised read counts and aggregation plots of are shown on top. (*G*) Per species, the pie chart consist of homologous genes of the 217 human MEL-differential genes with a topic 4 predicted region in their 200 kb up- and downstream locus (number of genes per species is shown between brackets). Pie charts show per species the number of these genes that do not have a MEL-predicted regions within their 200 kb up- and downstream extended gene locus; that have a MEL-predicted region within their extended locus which was either also found by liftOver; or not identified via liftOver. (*H*) Pearson’s correlation of deep layer scores between MEL-predicted regions near homologous MEL-specific genes between human and another species (‘Human-Species - Orthologous gene’), between MEL-predicted regions near different MEL-specific genes within one species (‘Species-Species - Different gene’), between MEL-predicted regions near the same MEL-specific gene within one species (‘Species-Species - Same gene’) and between non-MEL-predicted regions near homologous MEL-specific genes between human and a different species (‘Human-Species - Orthologous gene - noMEL’).

**
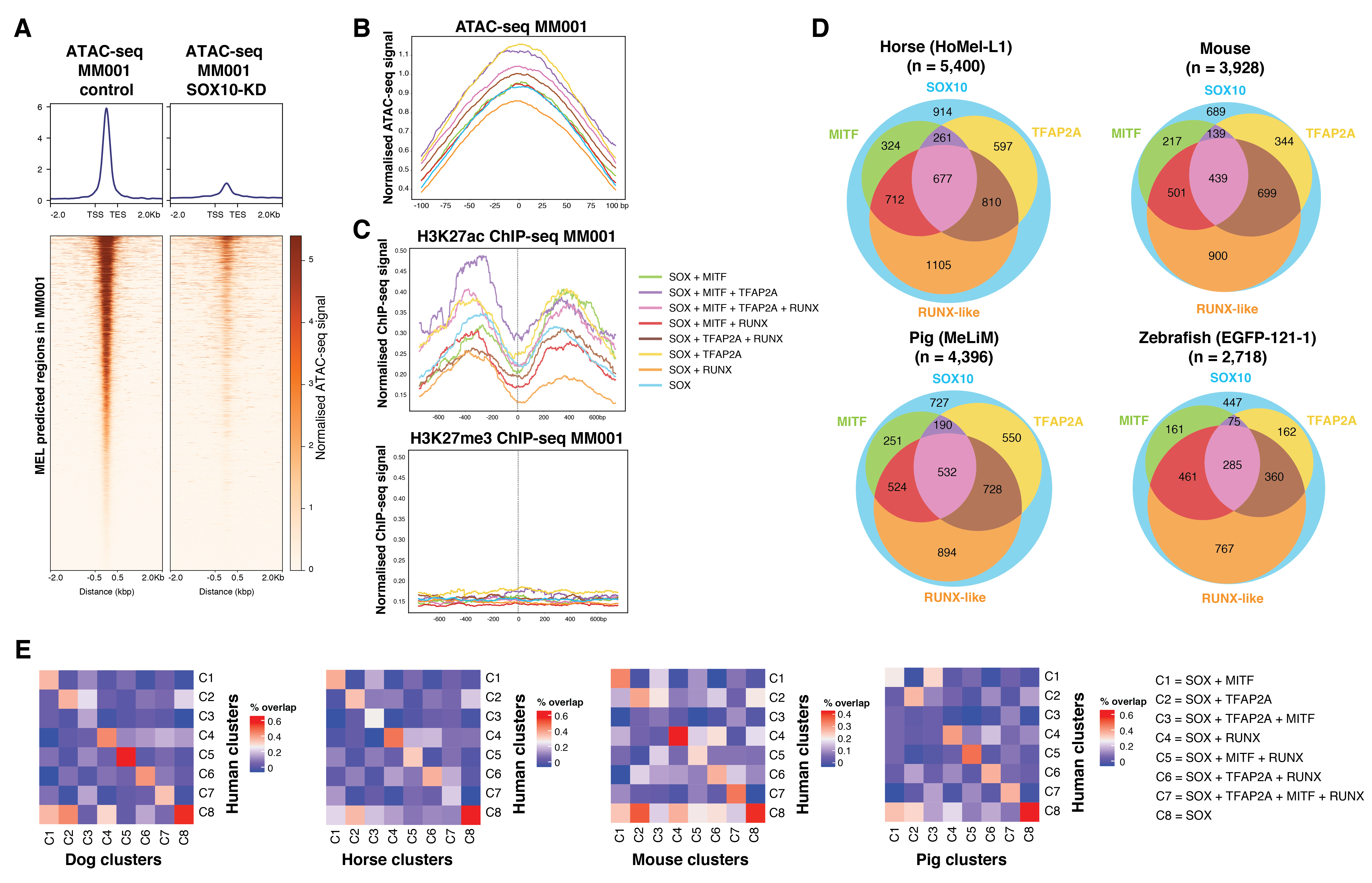
**

**Figure S6.** Conservation of COre Regulatory Complex clusters in MEL enhancers. (*A*) Heatmaps of ATAC-seq signal of MM001 after 72h of control knock-down (DMSO) and SOX10 knock-down on the 3,885 MEL predicted regions in MM001. Heatmaps are coloured by normalised read counts and ordered according to the ATAC-seq signal in the control sample and aggregation plots of are shown on top. (*B*) Aggregation plot of normalised ATAC-seq signal of MM001 on the human enhancer clusters. (*C*) Aggregation plots of H3K27ac and H3K27me3 ChIP-seq signal in MM001 on the human MEL enhancer clusters. (*D*) Venn diagrams of the TF motif-based enhancer clusters of DeepMEL-predicted MEL enhancers in horse, mouse, pig and zebrafish. The number of predicted MEL enhancers in each species is mentioned between brackets. (*E*) Heatmaps showing the correlation between MEL enhancers clusters in human and each of the other mammalian species. Heatmaps are coloured by the percentage of overlap between regions after liftOver of the enhancer coordinates to the human genome.





**Figure S7.** SOX10, TFAP2A, MITF and RUNX TF binding site motifs in relation to the nucleosome. (*A,B,C,D*) Nucleosome middle point (left), nucleosome start point (middle) and Tn5 cut site (right) on MEL-predicted regions containing one SOX10 motif (*A*), one TFAP2A motif (*B*), one MITF motif (*C*) and one RUNX-like motif (*D*) next to possible other motifs, where the regions are either centered on the ATAC-seq summit (grey) or on the specified motif (blue).


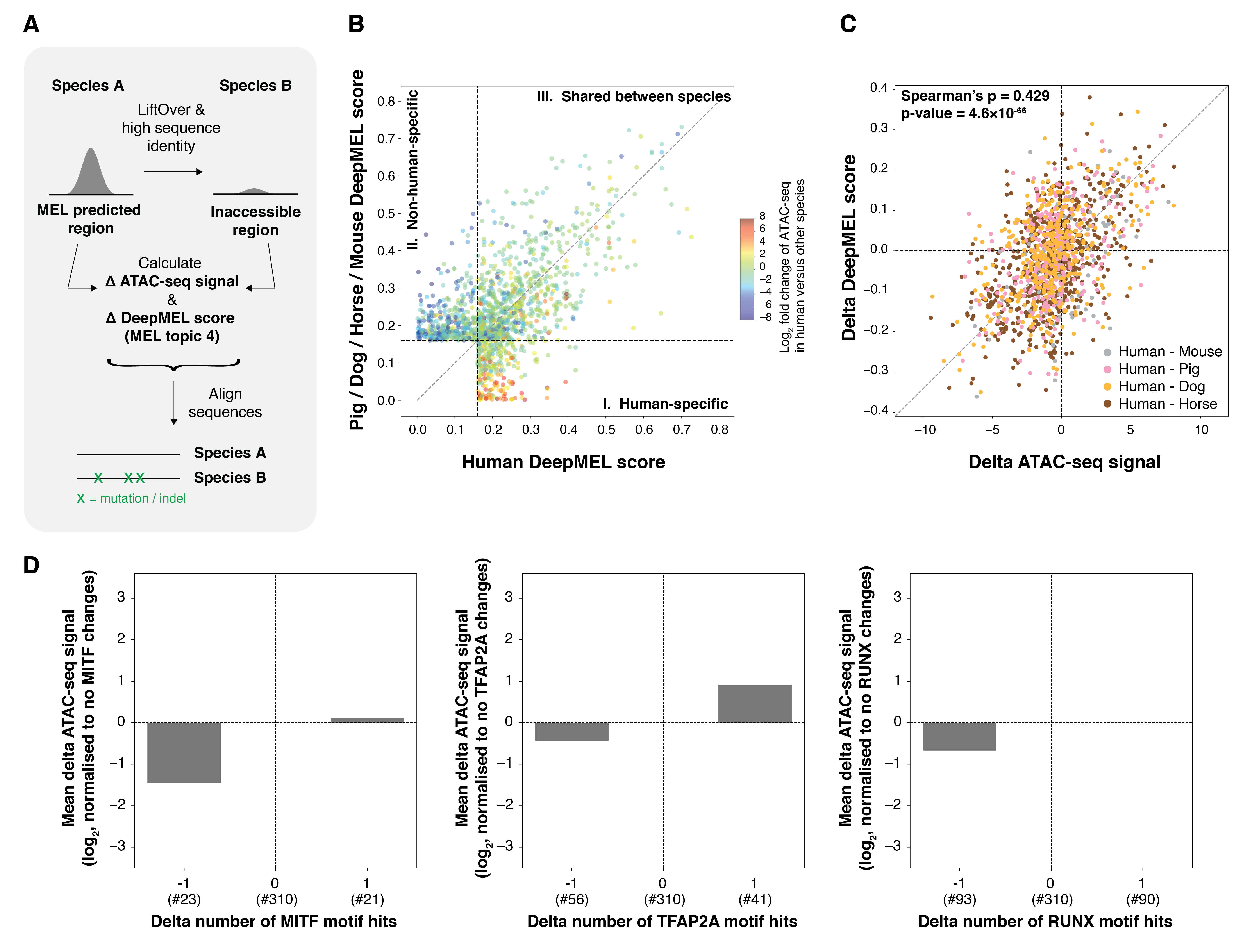


**Figure S8.** DeepMEL predicts evolutionary changes in accessibility. (*A*) Homologous (identified by stringent liftOver, minimum of 99% of the bases must remap) and high sequence identity (at least 80% of the bases must be identical) MEL enhancers that are accessible and predicted as MEL in only one species are used to identify causal *cis*-regulatory mutations by calculating the delta ATAC-seq signal and delta DeepMEL score for the MEL-specific topic (topic 4). Importantly, the change in accessibility can be due to either the collapse of MEL enhancer in species B, or *vice versa* gain of the MEL enhancer in species A. Both options have to be kept in mind as we are unable to verify whether the observed difference in accessibility originates from a loss- or gain-of-function. (*B*) Scatter plot of the DeepMEL prediction score for topic 4 in human and in another non-human mammalian species of pairs of homologous sequences. Only enhancers predicted as MEL-specific by DeepMEL (topic 4 score > 0.16) in at least one of the species are used here. Enhancers are represented by a dot and are coloured by the log_2_ fold change in ATAC-seq signal between human and the other species. In the first quadrant (I) enhancers that are predicted as MEL in human but not in the other species are shown; in quadrant (II) MEL enhancers of non-human species that are not predicted as MEL in human; and the third quadrant (III) contains enhancers that are MEL-predicted in both species. (*C*) Scatter plot of the delta ATAC-seq signal and delta DeepMEL prediction score for topic 4 of pairs of homologous enhancers between human and another mammalian species. Dots are colored depending on the species the human homolog was compared to. (*D*) Barplots showing the mean effect on the log_2_ delta ATAC-seq signal of a non-human region compared to the human homolog depending on the number of MITF, TFAP or RUNX motif hits lost or gained. Only regions having no change in the number of significant motifs hits for the other three CoRC factors were used. The y-axis is normalised to the category with no changes in the number of significant motif hits. The number of regions in each of the categories is mentioned (#).

**
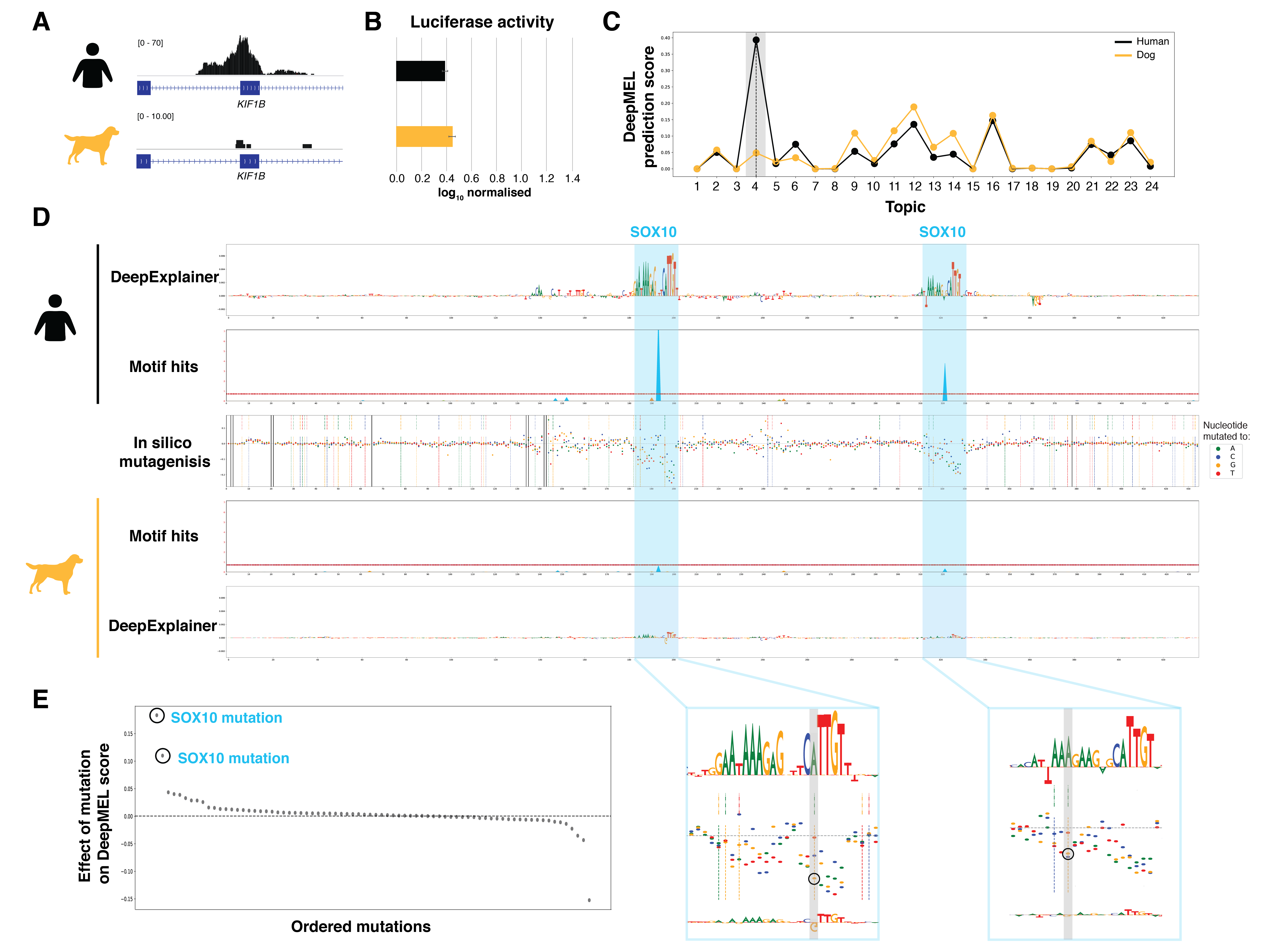
**

**Figure S9**. SOX10 binding site disruption destroys enhancer accessibility in *KIF1B* MEL enhancer. (*A*) Example MEL enhancer of KIF1B, which is accessible in human (MM001) but not in dog (Dog-OralMel-18249). (*B*) Both the human and dog regions show nearly no enhancer activity as measured by a luciferase assay in MM001. (*C*) The region is predicted as MEL in human but not in dog. (*D*) DeepExplainer profiles for human and dog are shown for the *KIF1B* region together with motifs hits for SOX10, TFAP2A, RUNX and MITF. Only for SOX10, two significant motif hits are visible in the human sequence. The middle row shows indels (black dashed lines) and individual point mutations (indicated by dashed lines, the colour indicates the human base (top dashed line) and the dog base (bottom dashed line)) and the dots represent the effect on the MEL DeepMEL score of the point mutations. In the zoom-in, the two A-to-G mutations that cause disruption of two SOX10 motif hits in the *KIF1B* region in dog are shown. (*E*) The effect on the MEL prediction score could mainly be explained by the two point mutations in the two SOX10 motifs.
