# Supplementary material for: Cross-species analysis of enhancer logic using deep learning
Source: Genome Res. 2020 Dec;30(12):1815–34. doi: 10.1101/gr.260844.120 (PMC7706731; doi:10.1101/gr.260844.120)
Supplement: Supplemental Material [file supp_gr.260844.120_Supplemental_Note.docx]

**Supplementary note**

*Optimisation of model hyperparameters of DeepMEL*

The hyperparameter optimisation was performed on topic 1 (promoters), topic 4 (MEL), and topic 7 (MES) by comparing the performance of DeepMEL (using the area under the receiver operator characteristic curve (auROC) and the precision recall curve (auPR)) on the training (all chromosomes except Chr11 and Chr2), validation (Chr11), and test (Chr2) sets. We explored the following parameters: size of the model (i.e. number of convolutional filters and neurons), size of the convolutional filters and length of the input DNA sequence (Fig. S3A-D).

In the original FactorNet (Quang and Xie 2019) architecture, the number of filters in the Conv1D layer, the number of neurons in TimeDistributed Dense layer, the number of neurons in Bidirectional LSTM layer and the number of neurons in Dense layer were 32, 32, 32 and 128, respectively. Calling these settings the 1×-sized model, we have tested models that were 1×, 2×, 4× and 8× the size (Fig. S3A). Performance of the different sized models were similar on topic 1 and topic 7. However, on topic 4, the 4×-sized model performed 7% better compared to other models in terms of auPR value (auPR of 0.35 for the 4×-sized model, compared to 0.31, 0.33, 0.32 for the 1×, 2 and 8× model, respectively).

For the filter size optimisation, DeepMEL was trained using filter sizes of 12, 16, 20, 24 and 28. Since the trained models performed very similarly (Fig. S3B), we selected the model with a filter size of 20, as this approximates the size of SOX10 dimers (~18bp).

Next, we trained DeepMEL using different lengths of input DNA: 30, 50, 100, 200, 400, 500, 600, 700, and 800 bp (Fig. S3C). The performance of the models increased with longer sequence lengths, until it plateaued at 400 bp. The 500 bp model was selected because it approximates the average peak size of the called peaks (around 500 bp).

In addition, we tested the performance of different model architectures. The architecture of deep learning models trained on genomics data falls generally into two main categories: CNNs that have more than one consecutive convolutional layers (DeepBind, DeepSEA, Basset) (Alipanahi et al. 2015; Zhou and Troyanskaya 2015; Kelley et al. 2016) and Hybrids (CNN-RNN) that have one convolutional layer and one recurrent layer (DanQ, FactorNet) (Quang and Xie 2016, 2019). We trained DeepMEL on both these architectures (Fig. S3D). The performance of the CNN and Hybrid model on the validation and test set was close to each other. However, the CNN model had a higher auROC and auPR on the training set, suggesting that the CNN model learned faster and that it may have a bias to the training set chromosomes. Therefore, in order to have an accurate and fair whole genome scoring, the Hybrid model was selected.

Lastly, during the hyperparameter and architecture optimisation above, we noticed that each time the model was trained for 2 epochs (since the performance on the validation set did not improve further). Therefore, we trained one more final model (this is the DeepMEL model used throughout the paper) by adding the validation set to the training set in order to have a larger training set, which was again trained for 2 epochs. As expected, the model with the larger training set performed better (Fig. S3E).
