# Supplementary material for: Cross-species analysis of enhancer logic using deep learning
Source: Genome Res. 2020 Dec;30(12):1815–34. doi: 10.1101/gr.260844.120 (PMC7706731; doi:10.1101/gr.260844.120)
Supplement: Supplemental Material [file supp_gr.260844.120_Supplemental_Code.zip › script_for_paper_BLS.html]

script\_for\_paper\_BLS


# Notebook to perform Branch Lenght Scoring (BLS)¶

### 1. Cluster-Buster with the icisTarget motif collection on conserved seqeunces per species¶

- Input: per species, merged fasta sequences of the conserved MEL enhancers were used.
- The icisTarget motif collection v8 was used, containing 20,003 motifs.
- Cluster-Buster will score each of these fastas with the 20,003 motifs.
- Per sequence, only the top CRM score is kept.

In [ ]:

```
module load Cluster-Buster/20180705-GCCcore-6.4.0

# Cluster-Buster output directory.
cbust_output_dir="/outdir";

# Run Cluster-Buster for each species.
for species in hg19 canFam3 mm10 susScr3 equCab2 danRer7; do
    # Specify FASTA file to score with the current species.
    species_regions_fasta_filename="Shared_Merged.${species}.fa";
    
    # Score all regions for one motif at the time.
    ls /staging/leuven/stg_00002/lcb/icistarget/data/motifCollection/v8/singletons_md5/*.cb \
        | cbust_output_dir="${cbust_output_dir}" \
            species="${species}" \
            species_regions_fasta_filename="${species_regions_fasta_filename}" \
            parallel \
                --env cbust_output_dir \
                --env species \
                --env species_regions_fasta_filename \
                -j 20 \
                '
                cb_filename={};
                cb_basename={/.};
                cb_basename="${cb_basename%.cb}";
             
                cbust_output_filename="${cbust_output_dir}/${cb_basename}.${species}.tsv";
             
                # Score current species FASTA file for current motif:
                #   - Minimum CRM score: 0.0
                #   - Use whole sequence of region (10000 bp up and down)
                #     to calculate background nucleotide frequencies.
                #   - Keep only top CRM score per region.
                # -r: # Range in bp for counting local nucleotide abundances (default = 100)
                # -t:  # Keep top X clusters per sequence (0 (= all))
                # -f:  #Output format: sorted by cluster score: seq name, score, seq number, rank
                # different than before: use minimal moritf score of 3
                cbust \
                    -c 0.0 \
                    -m 3 \
                    -r 10000 \
                    -t 1 \
                    -f 4 \
                    "${cb_filename}" \
                    "${species_regions_fasta_filename}" \
                    | cut -f 1,2 \
                    > "${cbust_output_filename}";
                '
             
done
```

### 2. BLS scoring¶

- Output from previous step: a file per motif per species containing the top CRM score for the motif in each of the conserved sequences.
- Combine CRM scores per region over multiple species for each motif into one output file per motif.
- Calculate the BLS per motif: outputs a tsv file with the summed BLS per motif.

In [ ]:

```
# 1. Combine CRM scores per region over multiple species for each motif into one output file per motif.

module load  Python/3.6.4-foss-2018a 

combine_CRM_scores_per_region_over_multiple_species.py \ # See script below
    --md5-motifs /staging/leuven/stg_00002/lcb/icistarget/data/motifCollection/v8/motifs_count_md5_to_motif_names.tsv \
    --regions Shared_Merged.region_ids \
    --o cbust/@motif@.crm_scores.tsv5 \
    --species hg19 canFam3 mm10 susScr3 equCab2 danRer7 \
    --input cbust/@motif@.@species@.tsv \
    --output cbust/@motif@.crm_scores.tsv \
    --jobs 10
```

In [ ]:

```
# 2. Calculate BLS per motif
    # with CRM min = 3

module load  Python/3.6.4-foss-2018a 

source /user/leuven/317/vsc31703/stg_00002/system/software/virtualenv/BioPython/bin/activate

calculate_branch_length_score_per_motif.py \
    --motifs  /user/leuven/317/vsc31703/stg_00002/Melanoma-species/motif_ids.tsv \
    --phylo hg19.100way.phyloP100way.newick \
    --species hg19 \
    --crm 3.0 \
    --input cbust/@motif@.crm_scores.tsv \
    --o cbust/bls.motifs.tsv \
    --jobs 10

deactivate
```

In [ ]:

```
# 3. Make HTML

/motif_ids_tsv_to_html.py \
-i bls.motifs.tsv \
-o bls.motifs.html \
-m 8 -n hgnc -d 1 -r -t 'Cross-species Melanoma'
```

### Extra Python scripts:¶

combine\_CRM\_scores\_per\_region\_over\_multiple\_species.py

In [ ]:

```
#!/usr/bin/env python3

import argparse
import sys
import os.path
import multiprocessing as mp
import numpy as np
import pandas as pd


def get_region_or_motif_ids(region_or_motif_ids_filename):
    """
    Read file with all region IDs or motif IDs in a list.
    """

    with open(region_or_motif_ids_filename, 'r') as region_or_motif_ids_fh:
        region_or_motif_ids_df = pd.read_csv(
            filepath_or_buffer=region_or_motif_ids_fh,
            sep='\t',
            header=None,
            names=['region_or_motif_id'],
            index_col=None,
            usecols=[0],
            comment='#',
            engine='c',
        )
        return region_or_motif_ids_df['region_or_motif_id'].tolist()

    return None


def get_motif_md5_to_motif_ids(motif_md5_to_motif_ids_filename):
    """
    Read file with all motif MD5 names to motif IDs in a dataframe.
    """

    with open(motif_md5_to_motif_ids_filename, 'r') as motif_md5_to_motif_ids_fh:
        motif_md5_to_motif_ids_df = pd.read_csv(
            filepath_or_buffer=motif_md5_to_motif_ids_fh,
            sep='\t',
            header=None,
            names=['motif_md5', 'motif_id'],
            index_col=0,
            usecols=[0, 1],
            comment='#',
            engine='c',
        )
        return motif_md5_to_motif_ids_df

    return None


def create_zerod_dataframe_for_cbust_of_species(region_ids, species):
    """
    Return a dataframe:
      - with all species names as columns.
      - with all region IDs as rows.
      - initialised to 0 (CRM score).
    so it can be filled in later with the actual values.
    """

    nbr_regions = len(region_ids)
    nbr_species = len(species)

    region_ids_to_crm_score_per_species_df = pd.DataFrame(
        np.zeros(
            (nbr_regions, nbr_species),
            dtype=np.float
        ),
        index=pd.Index(data=region_ids, name='region_ids'),
        columns=species,
    )

    return region_ids_to_crm_score_per_species_df


def read_cbust_output_format4(cbust_output_format4_filename):
    """
    Read Cluster-Buster output format 4 (-f 4) in a dataframe:
      - with region IDs ('region_id') as row names (column 1 of input file)
      - with CRM scores ('crm_score') as column names (column 2 of input file)
    and return it.
    """
    if not os.path.exists(cbust_output_format4_filename):
        print(
            'Error: Cluster-Buster output format 4 file "{0:s}" could not be found.'.format(
                    cbust_output_format4_filename
            ),
            file=sys.stderr,
        )
        sys.exit(1)

    with open(cbust_output_format4_filename, 'r') as cbust_output_format4_fh:
        region_ids_to_crm_score_df = pd.read_csv(
            filepath_or_buffer=cbust_output_format4_fh,
            sep='\t',
            header=None,
            names=['region_id', 'crm_score'],
            index_col=[0],
            usecols=[0, 1],
            comment='#',
            engine='c',
        )
        return region_ids_to_crm_score_df

    return None


def combine_crm_scores_per_region_over_multiple_species(
        region_ids,
        species,
        cbust_output_format4_filename_template,
        combined_crm_scores_filename_template,
        motif_md5_or_motif_id,
        motif_id):
    """
    Combine CRM scores per region over multiple species for this
    motif MD5 name (or motif ID).

      - region_ids:
          List of all region IDs written to the output file.
      - species:
          List of species names for which the CRM scores will
          be written to the output file.
      - cbust_output_format4_filename_template:
          Cluster-Buster output format 4 filename template.
          It should contain "@motif@" and "@species@" which are
          replaced by the value specified in "motif_md5_or_motif_id"
          and each species name specified in "species" respectively.
          Example: "/some/path/@motif@.@species@.tsv"
      - combined_crm_scores_filename_template:
          Combined CRM scores output filename template.
          It should contain "@motif@" which is replaced by the value
          specified in "motif_id".
          Example "/some/path/@motif@.crm_scores.tsv"
      - motif_md5_or_motif_id:
          motif MD5 name (or motif ID).
      - motif_id:
          motif ID.
    """

    print(motif_md5_or_motif_id)
    # Create a dataframe:
    #   - with all species names as columns.
    #   - with all region IDs as rows.
    #   - initialised to 0 (CRM score).
    region_ids_to_crm_score_per_species_df = create_zerod_dataframe_for_cbust_of_species(
        region_ids=region_ids,
        species=species
    )

    for species_name in species:
        # Replace "@motif@" and "@species@" in the cbust_output_format4_filename_template
        # with the current motif MD5 name and species name.
        current_cbust_output_format4_filename = cbust_output_format4_filename_template

        try:
            motif_md5_offset = current_cbust_output_format4_filename.rindex('@motif@')
        except ValueError:
            print(
                'Error: Cluster-Buster output format 4 filename template "{0:s}" does not contain "@motif@".'.format(
                    cbust_output_format4_filename_template
                ),
                file=sys.stderr,
            )
            sys.exit(1)

        current_cbust_output_format4_filename = current_cbust_output_format4_filename[
            :motif_md5_offset
        ] + motif_md5_or_motif_id + current_cbust_output_format4_filename[motif_md5_offset + 7:]

        try:
            species_offset = current_cbust_output_format4_filename.rindex('@species@')
        except ValueError:
            print(
                'Error: Cluster-Buster output format 4 filename template "{0:s}" does not contain "@species@".'.format(
                    cbust_output_format4_filename_template
                ),
                file=sys.stderr,
            )
            sys.exit(1)

        current_cbust_output_format4_filename = current_cbust_output_format4_filename[
            :species_offset
        ] + species_name + current_cbust_output_format4_filename[species_offset + 9:]

        region_ids_to_crm_score_df = read_cbust_output_format4(
            cbust_output_format4_filename=current_cbust_output_format4_filename
        )

        region_ids_to_crm_score_per_species_df.loc[region_ids_to_crm_score_df.index, species_name] = region_ids_to_crm_score_df.loc[:, 'crm_score']

    # Replace "@motif@" in the combined_crm_scores_filename_template with
    # current motif ID.
    combined_crm_scores_filename = combined_crm_scores_filename_template

    try:
        motif_md5_offset = combined_crm_scores_filename.rindex('@motif@')
    except ValueError:
        print(
            'Error: Combined CRM scores filename template "{0:s}" does not contain "@motif@".'.format(
                combined_crm_scores_filename_template
            ),
            file=sys.stderr,
        )
        sys.exit(1)

    combined_crm_scores_filename = combined_crm_scores_filename[
        :motif_md5_offset
    ] + motif_id + combined_crm_scores_filename[motif_md5_offset + 7:]

    print(combined_crm_scores_filename)
    # Write TSV with CRM scores for each region (or 0.0 if region does not have a CRM score)
    with open(combined_crm_scores_filename, 'w')  as combined_crm_scores_fh:
        region_ids_to_crm_score_per_species_df.to_csv(
            path_or_buf=combined_crm_scores_fh,
            sep='\t',
            float_format='%.10g',
            header=True,
            index=True,
        )


def main():
    parser = argparse.ArgumentParser(
        description='Combine CRM scores per region over multiple species for each motif to one output file per motif.'
    )

    motifs_mutually_exclusive_group = parser.add_mutually_exclusive_group(required=True)
    motifs_mutually_exclusive_group.add_argument(
        '-m',
        '--motifs',
        dest='motif_ids_filename',
        action='store',
        type=str,
        required=False,
        help="File with list of motif IDs."
    )
    motifs_mutually_exclusive_group.add_argument(
        '-5',
        '--md5-motifs',
        dest='motif_md5_to_motif_ids_filename',
        action='store',
        type=str,
        required=False,
        help="File with list of motif MD5 names to motif IDs."
    )

    parser.add_argument(
       '-r',
       '--regions',
       dest='region_ids_filename',
       action='store',
       type=str,
       required=True,
       help='File with list of region IDs.'
    )
    parser.add_argument(
        '-s',
        '--species',
        dest='species',
        action='store',
        type=str,
        nargs='+',
        required=True,
        help='List of species (assemblies).'
    )
    parser.add_argument(
        '-i',
        '--input',
        dest='cbust_output_format4_filename_template',
        action='store',
        type=str,
        required=True,
        help='Cluster-Buster (output format "-f 4") output files template: "@motif@" is replaced by the list of motifs provided by --motifs or by the motif MD5 name provided by --md5-motifs and "@species@" is replaced by the species provided with --species: "/some/path/@motif@.@species@.tsv"'
    )
    parser.add_argument(
        '-o',
        '--output',
        dest='combined_crm_scores_filename_template',
        action='store',
        type=str,
        required=True,
        help='Combined CRM scores output filename template: "@motif@" is replaced by the list of motifs provided by --motifs or by the motif IDs provided by --md5-motifs: "/some/path/@motif@.crm_scores.tsv"'
    )
    parser.add_argument(
        '-j',
        '--jobs',
        dest='nbr_processes',
        action='store',
        type=int,
        required=False,
        default=1,
        help='Specify the number of processes to use. Each job process will process files for one motif at the time. Default=1 Recommended=10'
    )


    args = parser.parse_args()

    # Get all region IDs.
    region_ids = get_region_or_motif_ids(
        region_or_motif_ids_filename=args.region_ids_filename
    )

    if args.motif_ids_filename:
        # Get all motif IDs.
        motif_ids = get_region_or_motif_ids(
            region_or_motif_ids_filename=args.motif_ids_filename
        )

        # Construct a fake motif MD5 to motif ID dataframe, where
        # the motif MD5 names are the same than the motif IDs.
        motif_md5_to_motif_ids_df = pd.DataFrame(
            np.array(motif_ids),
            index=pd.Index(motif_ids, name='motif_md5'),
            columns=['motif_id'],
        )
    else:
        # Get motif MD5 to motif IDs dataframe.
        motif_md5_to_motif_ids_df = get_motif_md5_to_motif_ids(
            motif_md5_to_motif_ids_filename=args.motif_md5_to_motif_ids_filename
        )


    # Create a multiprocess pool.
    pool = mp.Pool(args.nbr_processes)

    # Process files for each motif (motif MD5 or motif ID) in parallel.
    for motif_md5_or_motif_id in motif_md5_to_motif_ids_df.index:
        pool.apply_async(
            func=combine_crm_scores_per_region_over_multiple_species,
            args=(
                region_ids,
                args.species,
                args.cbust_output_format4_filename_template,
                args.combined_crm_scores_filename_template,
                motif_md5_or_motif_id,
                motif_md5_to_motif_ids_df.loc[motif_md5_or_motif_id, 'motif_id']
            )
        )

    pool.close()
    pool.join()

    sys.exit(0)


if __name__ == "__main__":
    main()
```

calculate\_branch\_length\_score\_per\_motif.py

In [ ]:

```
#!/usr/bin/env python3

import argparse
import functools
import multiprocessing as mp
import pandas as pd
import sys

import Bio.Phylo


def get_motif_ids(motif_ids_filename):
    """
    Read file with all motif IDs in a list.
    """

    with open(motif_ids_filename, 'r') as motif_ids_fh:
        motif_ids_df = pd.read_csv(
            filepath_or_buffer=motif_ids_fh,
            sep='\t',
            header=None,
            names=['motif_id'],
            index_col=None,
            usecols=[0],
            comment='#',
            engine='c',
        )
        return motif_ids_df['motif_id'].tolist()

    return None


@functools.lru_cache(maxsize=2048)
def get_total_branch_length_score_for_species(
        phylogenetic_tree_filename,
        for_species,
        species_for_tbls):
    """
    Get total branch lenght score for species specified in for_species:

      - phylogenetic_tree_filename:
          Phylogenetic tree filename in Newick format.
      - for_species:
          Species (assembly) of interest.
      - species_for_tbls:
          List of species to use to calculate the total branch length score.
          If it does not include species specified in "for_species" the
          total branch length score will be set to 0.0.
    """

    # Parse phylogenetic tree.
    phylogenetic_tree = Bio.Phylo.read(phylogenetic_tree_filename, 'newick')

    # Set of species names which will be used to calculate total branch length score.
    species_for_tbls = set(species_for_tbls)

    if for_species not in species_for_tbls:
        # If the species of interest is not in the set of species used to
        # calculate total branch length score, return 0.0 as it means the
        # motif is not available in our species of interest.
        return 0.0

    # Create phylogenetic tree with only the species defined in species_for_tbls.
    for species_to_prune in phylogenetic_tree.get_terminals():
        if species_to_prune.name not in species_for_tbls:
            phylogenetic_tree.prune(species_to_prune)

    # Check if all species in species_for_tbls were found.
    assert phylogenetic_tree.count_terminals() == len(species_for_tbls), \
        'Not all species of interest were found in the phylogenetic tree.'

    # Calculate the total branch length after only keeping those species of interest.
    return phylogenetic_tree.total_branch_length()


def get_total_branch_length_score_for_species_true_false_list(
        species_true_false_list,
        phylogenetic_tree_filename,
        for_species,
        all_species):
    """
    Get total branch lenght score for species specified in for_species:

      - species_true_false_list:
          List of True and False value, where each position in the list
          corresponds with the species (assembly) in all_species.
            - True:  CRM score for species (assembly) in list was > CRM scorethreshold.
            - False: CRM score for species (assembly) in list was <= CRM score threshold.
      - phylogenetic_tree_filename:
          Phylogenetic tree filename in Newick format.
      - for_species:
          Species (assembly) of interest.
      - all_species:
          List of all species (same length as species_true_false_list)

    This is a helper function to call "get_total_branch_length_score_for_species"
    for each row of "region_ids_to_true_false_per_species_df" dataframe
    with apply.
    """

    # Create list of species to use for total branch length score by selecting
    # all species which have a True value in species_true_false_list.
    species_for_tbls = tuple(
         true_false_all_species[1]
         for true_false_all_species in zip(species_true_false_list, all_species)
         if true_false_all_species[0]
    )

    # Calculate total branch length score for requested species.
    return get_total_branch_length_score_for_species(
        phylogenetic_tree_filename,
        for_species,
        species_for_tbls
    )


def read_crm_scores_per_region_over_multiple_species(
        crm_scores_per_region_over_multiple_species_filename):
    """
    Read CRM scores per region for multiple speecies in a dataframe:
      - with region IDs ('region_id') as row names (column 1 of input file)
      - with CRM scores for each species as column names (column 2 till n of input file)
    and return it.
    """

    with open(crm_scores_per_region_over_multiple_species_filename, 'r') as crm_scores_per_region_over_multiple_species_fh:
        region_ids_to_crm_scores_per_species_df = pd.read_csv(
            filepath_or_buffer=crm_scores_per_region_over_multiple_species_fh,
            sep='\t',
            header=0,
            index_col=[0],
            comment='#',
            engine='c',
        )
        return region_ids_to_crm_scores_per_species_df

    return None


def calculate_branch_length_score_for_motif(
        motif_id,
        crm_scores_per_region_over_multiple_species_filename,
        phylogenetic_tree_filename,
        for_species,
        crm_score_threshold):
    """
    Calculate branch length score for a motif for the species specified in for_species:

      - motif_id:
         Motif ID.
      - crm_scores_per_region_over_multiple_species_filename:
          Filename with CRM scores:
            - with region IDs ('region_id') as row names (column 1 of input file)
            - with CRM scores for each species as column names (column 2 till n of input file)
      - phylogenetic_tree_filename:
          Phylogenetic tree filename in Newick format.
      - for_species:
          Species (assembly) of interest.
      - crm_score_threshold:
          CRM score threshold which determines if a motif is present in
          a region for a species or not.
    """

    # Read CRM scores for regions scores in multiple species for a certain motif.
    region_ids_to_crm_scores_per_species_df = read_crm_scores_per_region_over_multiple_species(
        crm_scores_per_region_over_multiple_species_filename=crm_scores_per_region_over_multiple_species_filename
    )

    # Get list of all species for which we have CRM scores.
    all_species = tuple(region_ids_to_crm_scores_per_species_df.columns)

    # Filter out all regions for the species of interest (for_species)
    # that don't reach the CRM score threshold:
    #     region_ids_to_crm_scores_per_species_df[
    #          region_ids_to_crm_scores_per_species_df[for_species] > crm_score_threshold
    #     ]
    #
    # Then convert the filtered dataframe to a boolean dataframe:
    #   - True:  CRM score for current region ID in current species  > CRM score
    #   - False: CRM score for current region ID in current species <= CRM score
    region_ids_to_true_false_per_species_df = region_ids_to_crm_scores_per_species_df[
        region_ids_to_crm_scores_per_species_df[for_species] > crm_score_threshold
    ] > crm_score_threshold

    # If none of the CRM scores for the species of interest (for_species)
    # is above the CRM score threshold, return 0.0.
    if region_ids_to_true_false_per_species_df.shape[0] == 0:
        return motif_id, 0.0

    # Calculate the total branch length score for each region and sum those
    # branch length scores for each region so we have a total score for the
    # current motif.
    return (
        motif_id,
        region_ids_to_true_false_per_species_df.apply(
            func=get_total_branch_length_score_for_species_true_false_list,
            axis=1,
            args=(
                phylogenetic_tree_filename,
                for_species,
                all_species
            )
        ).sum()
    )


def main():
    parser = argparse.ArgumentParser(
        description='Calculate branch length score for each motif.'
    )
    parser.add_argument(
        '-m',
        '--motifs',
        dest='motif_ids_filename',
        action='store',
        type=str,
        required=True,
        help="File with list of motif IDs."
    )
    parser.add_argument(
        '-p',
        '--phylo',
        dest='phylogenetic_tree_filename',
        action='store',
        type=str,
        required=True,
        help='Phylogenetic tree file in Newick format.'
    )
    parser.add_argument(
        '-s',
        '--species',
        dest='for_species',
        action='store',
        type=str,
        required=True,
        help='Species (assembly) of interest for which to calculate the branch length score for each motif.'
    )
    parser.add_argument(
        '-c',
        '--crm',
        dest='crm_score_threshold',
        action='store',
        type=float,
        required=False,
        default=3.0,
        help='CRM score threshold to use to decide if a CRM is found in a region or not. Default: 3.0'
    )
    parser.add_argument(
        '-i',
        '--input',
        dest='crm_scores_per_region_over_multiple_species_filename_template',
        action='store',
        type=str,
        required=True,
        help='CRM scores per region over multiple species filename template: "@motif@" is replaced by the list of motifs provided by --motifs: "/some/path/@motif@.crm_scores.tsv"'
    )
    parser.add_argument(
        '-o',
        '--output',
        dest='bls_filename',
        action='store',
        type=str,
        required=True,
        help='Output filename with a branch length score for each motif.'
    )
    parser.add_argument(
        '-j',
        '--jobs',
        dest='nbr_processes',
        action='store',
        type=int,
        required=False,
        default=1,
        help='Specify the number of processes to use. Each job process will process files for one motif at the time. Default=1 Recommended=10'
    )

    args = parser.parse_args()

    # Get all motif IDs.
    motif_ids = get_motif_ids(motif_ids_filename=args.motif_ids_filename)


    # Create a multiprocess pool.
    pool = mp.Pool(args.nbr_processes)

    bls_scores_results = list()

    # Process files for each motif (motif MD5 or motif ID) in parallel.
    for motif_id in motif_ids:
        # Replace "@motif@" in crm_scores_per_region_over_multiple_species_filename_template
        # with the current motif MD5 name and species name.
        crm_scores_per_region_over_multiple_species_filename = args.crm_scores_per_region_over_multiple_species_filename_template

        try:
            motif_id_offset = crm_scores_per_region_over_multiple_species_filename.rindex('@motif@')
        except ValueError:
            print(
                'Error: CRM scores per region over multiple species filename template "{0:s}" does not contain "@motif@".'.format(
                    args.crm_scores_per_region_over_multiple_species_filename_template
                ),
                file=sys.stderr,
            )
            sys.exit(1)

        crm_scores_per_region_over_multiple_species_filename = crm_scores_per_region_over_multiple_species_filename[
            :motif_id_offset
        ] + motif_id + crm_scores_per_region_over_multiple_species_filename[motif_id_offset + 7:]

        bls_scores_results.append(
            pool.apply_async(
                func=calculate_branch_length_score_for_motif,
                args=(
                    motif_id,
                    crm_scores_per_region_over_multiple_species_filename,
                    args.phylogenetic_tree_filename,
                    args.for_species,
                    args.crm_score_threshold
                )
            )
        )

    pool.close()
    pool.join()

    with open(args.bls_filename, 'w') as bls_fh:
        for bls_scores_result in bls_scores_results:
            # Write motif name and branch length score for motif.
            print(
                *bls_scores_result.get(),
                sep='\t',
                file=bls_fh
            )

    sys.exit(0)


if __name__ == "__main__":
    main()
```

motif\_ids\_tsv\_to\_html.py

In [ ]:

```
#!/usr/bin/env python3

import argparse
import html
import os
import sys


if os.path.exists('/home/icistarget/data/'):
    data_dir = '/home/icistarget/data'
elif os.path.exists('/staging/leuven/stg_00002/lcb/icistarget/data/'):
    data_dir = '/staging/leuven/stg_00002/lcb/icistarget/data'
else:
    data_dir = os.path.join(os.path.dirname(__file__), 'data')

motif_collections_dir = os.path.join(data_dir, 'motifCollection')
motif_to_tf_dir = os.path.join(data_dir, 'motif2tf/snapshots')


class MotifCollections:
    zips = {
        3: os.path.join(motif_collections_dir, 'zips/motifs-v3.zip'),
        6: os.path.join(motif_collections_dir, 'zips/motifs-v6-nr.zip'),
        7: os.path.join(motif_collections_dir, 'zips/motif_collection_v7.zip'),
        8: os.path.join(motif_collections_dir, 'zips/motif_collection_v8.zip'),
        9: os.path.join(motif_collections_dir, 'zips/motif_collection_v9.zip'),
    }

    extracted = {
        3: os.path.join(motif_collections_dir, 'v3'),
        6: os.path.join(motif_collections_dir, 'v6'),
        7: os.path.join(motif_collections_dir, 'v7'),
        8: os.path.join(motif_collections_dir, 'v8'),
        9: os.path.join(motif_collections_dir, 'v9'),
    }

    motifs_count_md5_to_motif_names_filename = {
        7: os.path.join(motif_collections_dir, 'v7/motifs_count_md5_to_motif_names.tsv'),
        8: os.path.join(motif_collections_dir, 'v8/motifs_count_md5_to_motif_names.tsv'),
        9: os.path.join(motif_collections_dir, 'v9/motifs_count_md5_to_motif_names.tsv'),
    }

    @staticmethod
    def get_motifs_count_md5_to_motif_names_filename(motif_collection_version):
        return MotifCollections.motifs_count_md5_to_motif_names_filename.get(motif_collection_version)

    @staticmethod
    def get_motifs_count_md5_to_motif_names(motif_collection_version):
        motifs_count_md5_to_motif_id = dict()
        motifs_count_md5_to_alternative_motif_ids = dict()

        motifs_count_md5_to_motif_names_filename = MotifCollections.get_motifs_count_md5_to_motif_names_filename(
            motif_collection_version=motif_collection_version,
        )

        if motifs_count_md5_to_motif_names_filename:
            with open(motifs_count_md5_to_motif_names_filename, 'r') as fh:
                for line in fh:
                    line = line.rstrip('\n')

                    if line.startswith('#'):
                        continue

                    columns = line.split('\t')

                    if len(columns) >= 2:
                        # motif_m5  motif_id  alternative_motif_ids
                        motif_md5 = columns[0]
                        motif_id = columns[1]
                        alternative_motif_ids = columns[2:]

                        motifs_count_md5_to_motif_id[motif_md5] = motif_id
                        motifs_count_md5_to_alternative_motif_ids[motif_md5] = alternative_motif_ids

        return motifs_count_md5_to_motif_id, motifs_count_md5_to_alternative_motif_ids


class MotifToTF:
    filenames = {
        (3, 'flybase', 'direct'): os.path.join(motif_to_tf_dir, 'motifs-v3-flybase-m0.0-o0.0.tbl'),
        (6, 'flybase', 'direct'): os.path.join(motif_to_tf_dir, 'motifs-v6-flybase-m0.000-o0.0.tbl'),
        (3, 'flybase', 'indirect'): os.path.join(motif_to_tf_dir, 'motifs-v6-flybase-m0.001-o0.0.tbl'),
        (6, 'hgnc', 'direct'): os.path.join(motif_to_tf_dir, 'motifs-v6-nr-hgnc-m0.0-o0.0.tbl'),
        (6, 'hgnc', 'indirect'): os.path.join(motif_to_tf_dir, 'motifs-v6-nr-hgnc-m0.001-o0.0.tbl'),
        (6, 'mgi', 'indirect'): os.path.join(motif_to_tf_dir, 'motifs-v6-nr.mgi-m0.001-o0.0.tbl'),
        (7, 'fbgn', 'direct'): os.path.join(motif_to_tf_dir, 'motifs-v7-nr.fbgn-m0.000-o0.0.tbl'),
        (7, 'fbgn', 'indirect'): os.path.join(motif_to_tf_dir, 'motifs-v7-nr.fbgn-m0.001-o0.0.tbl'),
        (7, 'flybase', 'direct'): os.path.join(motif_to_tf_dir, 'motifs-v7-nr.flybase-m0.000-o0.0.tbl'),
        (7, 'flybase', 'indirect'): os.path.join(motif_to_tf_dir, 'motifs-v7-nr.flybase-m0.001-o0.0.tbl'),
        (7, 'hgnc', 'direct'): os.path.join(motif_to_tf_dir, 'motifs-v7-nr.hgnc-m0.000-o0.0.tbl'),
        (7, 'hgnc', 'indirect'): os.path.join(motif_to_tf_dir, 'motifs-v7-nr.hgnc-m0.001-o0.0.tbl'),
        (7, 'mgi', 'direct'): os.path.join(motif_to_tf_dir, 'motifs-v7-nr.mgi-m0.000-o0.0.tbl'),
        (7, 'mgi', 'indirect'): os.path.join(motif_to_tf_dir, 'motifs-v7-nr.mgi-m0.001-o0.0.tbl'),
        (8, 'fbgn', 'direct'): os.path.join(motif_to_tf_dir, 'motifs-v8-nr.fbgn-m0.000-o0.0.tbl'),
        (8, 'fbgn', 'indirect'): os.path.join(motif_to_tf_dir, 'motifs-v8-nr.fbgn-m0.001-o0.0.tbl'),
        (8, 'flybase', 'direct'): os.path.join(motif_to_tf_dir, 'motifs-v8-nr.flybase-m0.000-o0.0.tbl'),
        (8, 'flybase', 'indirect'): os.path.join(motif_to_tf_dir, 'motifs-v8-nr.flybase-m0.001-o0.0.tbl'),
        (8, 'hgnc', 'direct'): os.path.join(motif_to_tf_dir, 'motifs-v8-nr.hgnc-m0.000-o0.0.tbl'),
        (8, 'hgnc', 'indirect'): os.path.join(motif_to_tf_dir, 'motifs-v8-nr.hgnc-m0.001-o0.0.tbl'),
        (8, 'mgi', 'direct'): os.path.join(motif_to_tf_dir, 'motifs-v8-nr.mgi-m0.000-o0.0.tbl'),
        (8, 'mgi', 'indirect'): os.path.join(motif_to_tf_dir, 'motifs-v8-nr.mgi-m0.001-o0.0.tbl'),
        (9, 'fbgn', 'direct'): os.path.join(motif_to_tf_dir, 'motifs-v9-nr.fbgn-m0.000-o0.0.tbl'),
        (9, 'fbgn', 'indirect'): os.path.join(motif_to_tf_dir, 'motifs-v9-nr.fbgn-m0.001-o0.0.tbl'),
        (9, 'flybase', 'direct'): os.path.join(motif_to_tf_dir, 'motifs-v9-nr.flybase-m0.000-o0.0.tbl'),
        (9, 'flybase', 'indirect'): os.path.join(motif_to_tf_dir, 'motifs-v9-nr.flybase-m0.001-o0.0.tbl'),
        (9, 'hgnc', 'direct'): os.path.join(motif_to_tf_dir, 'motifs-v9-nr.hgnc-m0.000-o0.0.tbl'),
        (9, 'hgnc', 'indirect'): os.path.join(motif_to_tf_dir, 'motifs-v9-nr.hgnc-m0.001-o0.0.tbl'),
        (9, 'mgi', 'direct'): os.path.join(motif_to_tf_dir, 'motifs-v9-nr.mgi-m0.000-o0.0.tbl'),
        (9, 'mgi', 'indirect'): os.path.join(motif_to_tf_dir, 'motifs-v9-nr.mgi-m0.001-o0.0.tbl'),
    }

    @staticmethod
    def get_motif_to_tf_filename(motif_to_tf_version, nomenclature, direct_or_indirect):
        return MotifToTF.filenames.get((motif_to_tf_version, nomenclature, direct_or_indirect))

    @staticmethod
    def get_motif_to_tf(motif_to_tf_version, nomenclature, direct_or_indirect):
        motif_to_tf = dict()

        motif_to_tf_filename = MotifToTF.filenames.get((motif_to_tf_version, nomenclature, direct_or_indirect))

        if motif_to_tf_filename:
            with open(motif_to_tf_filename, 'r') as fh:
                for line in fh:
                    line = line.rstrip('\n')

                    if line.startswith('#'):
                        continue

                    columns = line.split('\t')

                    if len(columns) == 4:
                        # motif_id  motif_description  gene_name  description
                        motif_id = columns[0]
                        tf = columns[2]
                        motif_to_tf.setdefault(motif_id, []).append(tf)
                    if len(columns) == 13:
                        # motif_id  motif_name  motif_description  source_name  source_version  gene_name
                        # motif_similarity_qvalue  similar_motif_id  similar_motif_description
                        # orthologous_identity  orthologous_gene_name  orthologous_species  description
                        motif_id = columns[0]
                        tf = columns[5]
                        motif_to_tf.setdefault(motif_id, []).append(tf)

        return motif_to_tf


def convert_tsv_to_html(motifs_tsv_filename, motifs_html_filename, motif_collection_version,
                        motif_to_tf_direct, motif_to_tf_indirect,
                        motif_id_column, motif_md5_column,
                        motifs_count_md5_to_motif_id, motifs_count_md5_to_alternative_motif_ids,
                        column_indices_output, display_rank, title):
    """
    Convert a TSV file with a motif ID column to an HTML report with:
      - Rank (if display_rank is True)
      - motif ID
      - alternative motif IDs (if motif_md5_column was chosen)
      - motif logo
      - direct TFs
      - indirect TFs
    columns added.

    :param motifs_tsv_filename: TSV file with motif ID column.
    :param motifs_html_filename: HTML file to which the report is written.
    :param motif_collection_version: Motif collection version of the used motif IDs.
    :param motif_to_tf_direct: motif to TF dictionary with all directly annotated TFs.
    :param motif_to_tf_indirect: motif to TF dictionary with all directly and indirectly annotated TFs.
    :param motif_id_column: column index that contains the motif IDs in the TSV file.
    :param motif_md5_column: column index that contains the motif md5 name in the TSV file.
    :param motifs_count_md5_to_motif_id: motif md5 name to motif ID dictionary.
    :param motifs_count_md5_to_alternative_motif_ids: motif md5 name to alternative motif ID dictionary.
    :param column_indices_output: Only output those column indices of the TSV file to the report (None: output all columns).
    :param display_rank: Display the rank for each row (True/False).
    :param title: Title used in the HTML report.
    :return: 
    """

    # Specify jQuery version.
    jquery_js_url = 'https://cdnjs.cloudflare.com/ajax/libs/jquery/3.2.1/jquery.min.js'

    # Compose DataTables version on https://datatables.net/download/
    #   - jQuery:
    #       - No jQuery
    #   - Styling:
    #       - DataTables
    #   - DataTables:
    #       - DataTables
    #   - Buttons:
    #       - Buttons
    #       - Column visibility
    #       - HTML5 export
    #       - JSZip
    #       - Print view
    #   - ColReorder:
    #       - ColReorder
    #   - FixedHeader:
    #       - FixedHeader
    #   - RowReorder:
    #       - RowReorder
    #   - Select:
    #       - Select
    #   - Packaging options:
    #       - Minify
    #       - Single file
    #       - CDN
    data_tables_js_url = 'https://cdn.datatables.net/v/dt/jszip-3.1.3/dt-1.10.15/b-1.3.1/b-colvis-1.3.1/b-html5-1.3.1/b-print-1.3.1/cr-1.3.3/fh-3.1.2/rr-1.2.0/se-1.2.2/datatables.min.js'
    data_tables_css_url = 'https://cdn.datatables.net/v/dt/jszip-3.1.3/dt-1.10.15/b-1.3.1/b-colvis-1.3.1/b-html5-1.3.1/b-print-1.3.1/cr-1.3.3/fh-3.1.2/rr-1.2.0/se-1.2.2/datatables.min.css'

    with open(motifs_html_filename, 'w') as fh_html:
        print(
            '''<!DOCTYPE html>
<html>
<head>
  <meta charset="utf-8">''',
            '''  <title>{0:s}</title>'''.format(html.escape(title)),
            '''  <script type="text/javascript" charset="utf-8" src="{0:s}"></script>'''.format(jquery_js_url),
            '''  <script type="text/javascript" charset="utf-8" src="{0:s}"></script>'''.format(data_tables_js_url),
'''  <script>
    $(document).ready( function () {
        // DataTable
        var table = $('#motif_table_id').DataTable( {
            //dom: '<B><f><lp>rtip',
            dom: '<"clearfix"B><"clearfix"f><"clearfix"lp><"clearfix"i>rtip',
            ordering: true,
            searching: true,
            paging: true,
            lengthMenu: [
                [50, 100, 200, 300, 400, 500, 600, 700, 800, 900, 1000, -1],
                [50, 100, 200, 300, 400, 500, 600, 700, 800, 900, 1000, "All"],
            ],
            pageLength: 500,
            buttons: [
                {
                    extend: 'colvis',
                    text: 'Show/hide columns',
                },
                {
                    extend: 'collection',
                    text: 'Copy',
                    buttons: [
                        {
                            extend: 'copyHtml5',
                            text: 'Selected',
                            exportOptions: {
                                 columns: ':visible',
                                 modifier: {
                                     selected: true,
                                 }
                            }
                        },
                        {
                            extend: 'copyHtml5',
                            text: 'All',
                            exportOptions: {
                                 columns: ':visible',
                            }
                        },
                    ],
                },
                {
                    extend: 'collection',
                    text: 'Export',
                    buttons: [
                        {
                            extend: 'collection',
                            text: 'CSV',
                            buttons: [
                                {
                                    extend: 'csvHtml5',
                                    text: 'Selected',
                                    exportOptions: {
                                         columns: ':visible',
                                         modifier: {
                                             selected: true,
                                         }
                                    }
                                },
                                {
                                    extend: 'csvHtml5',
                                    text: 'All',
                                    exportOptions: {
                                         columns: ':visible',
                                    }
                                },
                            ],
                        },
                        {
                            extend: 'collection',
                            text: 'Excel',
                            buttons: [
                                {
                                    extend: 'excelHtml5',
                                    text: 'Selected',
                                    exportOptions: {
                                         columns: ':visible',
                                         modifier: {
                                             selected: true,
                                         }
                                    }
                                },
                                {
                                    extend: 'excelHtml5',
                                    text: 'All',
                                    exportOptions: {
                                         columns: ':visible',
                                    }
                                },
                            ],
                        },
                    ],
                },
                {
                    extend: 'collection',
                    text: 'Print',
                    buttons: [
                        {
                            extend: 'print',
                            text: 'Selected',
                            exportOptions: {
                                 stripHtml: false,
                                 columns: ':visible',
                                 modifier: {
                                     selected: true,
                                 }
                            }
                        },
                        {
                            extend: 'print',
                            text: 'All',
                            exportOptions: {
                                 stripHtml: false,
                                 columns: ':visible',
                            }
                        },
                    ],
                },
            ],
            colReorder: true,
            fixedHeader: {
                footer: true,
            },
            select: true,
            rowReorder: true,
        } );

        // Add search filters to each column of the table footer.
        table.columns().every( function () {
            var column = this;

            $('input', this.footer() ).on('keyup change', function () {
                column
                    .search(this.value)
                    .draw();
            } );
        } );

    } );
  </script>''',
            '  <link rel="stylesheet" type="text/css" href="{0:s}">'.format(data_tables_css_url),
            '''  <style>
      body {
          margin: 10px 30px;
      }
      .clearfix:after {
          content: "";
          clear: both;
          display: table;
          margin: 10px 0;
      }
      a.dt-button {
          margin: 10px 15px 10px 0;
      }
      a.buttons-colvis {
          margin: 10px 50px 10px 0;
      }
      #motif_table_id_info {
          padding-left: 20px;
          color: blue;
      }
      img.logo {
          width: 200px;
      }
  </style>
  </head>
  <body>
    <table id="motif_table_id" class="display compact">''',
            sep='\n',
            file=fh_html,
        )

        header_line = False
        header_printed = False
        footer=''
        current_ranking = 0

        with open(motifs_tsv_filename, 'r') as fh_tsv:
            for line in fh_tsv:
                line = line.rstrip('\r\n')

                if not line:
                    continue

                if line.startswith('#'):
                    # Get header and remove leading comment and whitespace.
                    header_line = True
                    line = line.lstrip('# ')
                else:
                    header_line = False
                    current_ranking += 1

                columns = line.split('\t')

                if column_indices_output:
                    # Take only specified columns for displaying in the output after the motif info.
                    output_columns = [columns[column_idx] for column_idx in column_indices_output]
                else:
                    # Make a copy of all columns.
                    output_columns = columns.copy()

                    # Make the corresponding column indices output list,
                    # which will be used for naming the columns if no header was found.
                    column_indices_output = list(range(len(columns)))

                    if motif_id_column is not None:
                        # Remove the motif ID column from the output list as
                        # it will be displayed in the motif info part already.
                        output_columns.pop(motif_id_column)
                        column_indices_output.remove(motif_id_column)

                if header_line is True:
                    # Print table header with header names used in the TSV file.
                    print(
                        '<thead>',
                        ('<tr>' +
                         ('<th>rank</th>'
                          if display_rank
                          else '') +
                         '<th>motif_id</th>' +
                         ('<th>alternative_motif_ids</th>'
                          if motif_md5_column is not None
                          else '') +
                         '<th>motif logo</th>'
                         '<th>direct TFs</th>'
                         '<th>indirect TFs</th>' +
                         ('<th>' + '</th><th>'.join([html.escape(output_column)
                                                    for output_column in output_columns]
                                                    ) + '</th>'
                          if len(output_columns) > 0
                          else '') +
                         '</tr>'),
                        '</thead>',
                        '<tbody>',
                        sep='\n',
                        file=fh_html,
                    )

                    # Make table footer with search boxes for each column.
                    footer = ('<tfoot>\n' +
                              ('<tr>' +
                               ('<th><input type="text" placeholder="Search rank" /></th>'
                                if display_rank
                                else '') +
                               '<th><input type="text" placeholder="Search motif_id" /></th>' +
                               ('<th><input type="text" placeholder="Search alternative_motif_ids" /></th>'
                                if motif_md5_column is not None
                                else '') +
                               '<th><input type="text" placeholder="Search motif logo" /></th>'
                               '<th><input type="text" placeholder="Search direct TFs" /></th>'
                               '<th><input type="text" placeholder="Search indirect TFs" /></th>' +
                               ('<th><input type="text" placeholder="Search ' +
                                '" /></th><th><input type="text" placeholder="Search '.join(
                                    [html.escape(output_column)
                                     for output_column in output_columns]
                                ) + '" /></th>'
                                if len(output_columns) > 0
                                else '') +
                               '</tr>') +
                              '</tfoot>')

                    header_printed = True

                    continue
                elif current_ranking == 1 and header_printed is False:
                    # Print table header with header column numbered names.
                    print(
                        '<thead>',
                        ('<tr>' +
                         ('<th>Rank</th>'
                          if display_rank
                          else '') +
                         '<th>motif_id</th>' +
                         ('<th>alternative_motif_ids</th>'
                          if motif_md5_column is not None
                          else '') +
                         '<th>motif logo</th>'
                         '<th>direct TFs</th>'
                         '<th>indirect TFs</th>' +
                         ('<th>' + '</th><th>'.join(['column {0:d}'.format(column_idx + 1)
                                                    for column_idx in column_indices_output]
                                                    ) + '</th>'
                          if len(output_columns) > 0
                          else '') +
                         '</tr>'),
                        '</thead>',
                        '<tbody>',
                        sep='\n',
                        file=fh_html
                    )

                    header_printed = True

                    # Make table footer with search boxes for each column.
                    footer = ('<tfoot>\n' +
                              ('<tr>' +
                               ('<th><input type="text" placeholder="Search rank" /></th>'
                                if display_rank
                                else '') +
                               '<th><input type="text" placeholder="Search motif_id" /></th>' +
                               ('<th><input type="text" placeholder="Search alternative_motif_ids" /></th>'
                                if motif_md5_column is not None
                                else '') +
                               '<th><input type="text" placeholder="Search motif logo" /></th>'
                               '<th><input type="text" placeholder="Search direct TFs" /></th>'
                               '<th><input type="text" placeholder="Search indirect TFs" /></th>' +
                               ('<th><input type="text" placeholder="Search ' +
                                '" /></th><th><input type="text" placeholder="Search '.join(
                                    ['column {0:d}'.format(column_idx + 1)
                                     for column_idx in column_indices_output]
                                ) + '" /></th>'
                                if len(output_columns) > 0
                                else '') +
                               '</tr>') +
                              '</tfoot>')

                if motif_md5_column is not None:
                    # Get motif md5 name.
                    motif_md5 = columns[motif_md5_column]
                    # Get motif ID corresponding to motif md5 name.
                    motif_id = motifs_count_md5_to_motif_id[motif_md5]
                    # Get alternative motif IDs corresponding to motif md5 name.
                    alternative_motif_ids = motifs_count_md5_to_alternative_motif_ids[motif_md5]
                else:
                    # Get motif ID.
                    motif_id = columns[motif_id_column]

                # Make URL for motif logo.
                motif_id_logo_url = 'http://motifcollections.aertslab.org/v{0:d}/logos/{1:s}.png'.format(
                    motif_collection_version,
                    motif_id,
                )

                # Print each row of the table.
                print(
                    ('<tr>' +
                     ('<td>' + str(current_ranking) + '</td>'
                      if display_rank
                      else '') +
                     '<td>' + html.escape(motif_id) + '</td>' +
                     ('<td>' + html.escape(' '.join(alternative_motif_ids)) + '</td>'
                      if motif_md5_column is not None
                      else '') +
                     '<td><img src="' + motif_id_logo_url + '" alt="' + html.escape(motif_id) + '" class="logo" /></td>'
                     '<td>' + ' '.join([html.escape(tf_direct)
                                        for tf_direct in motif_to_tf_direct.get(motif_id, ' ')]
                                       ) + '</td>'
                     '<td>' + ' '.join([html.escape(tf_indirect)
                                        for tf_indirect in motif_to_tf_indirect.get(motif_id, ' ')]
                                       ) + '</td>' +
                     ('<td>' + '</td><td>'.join(output_columns) + '</td>'
                      if len(output_columns) > 0
                      else '') +
                     '</tr>'),
                    sep='\n',
                    file=fh_html,
                )

        print(
            '</tbody>',
            footer,
            '</table>',
            '</html>',
            sep='\n',
            file=fh_html,
        )


def main():
    parser = argparse.ArgumentParser(
        description='Convert TSV with motif IDs to a HTML file with logos and TF annotation.'
    )
    parser.add_argument(
        '-i',
        '--tsv',
        dest='tsv_filename',
        action='store',
        type=str,
        required=True,
        help='TSV input file with motifs'
    )
    parser.add_argument(
        '-o',
        '--html',
        dest='html_filename',
        action='store',
        type=str,
        required=True,
        help='HTML output file'
    )
    parser.add_argument(
        '-m',
        '--motif-collection-version',
        dest='motif_collection_version',
        action='store',
        type=int,
        required=True,
        choices={3, 6, 7, 8, 9},
        default=9,
        help='Motif collection version: 3, 6, 7, 8, 9 (default: 9)'
    )
    parser.add_argument(
        '-n',
        '--nomenclature',
        dest='nomenclature',
        action='store',
        type=str,
        required=True,
        choices={'fbgn', 'flybase', 'hgnc', 'mgi'},
        help='Nomenclature to use for motif to TF: fbgn, flybase, hgnc, mgi'
    )

    motif_group = parser.add_mutually_exclusive_group(required=True)
    motif_group.add_argument(
        '-d',
        '--motif-id-column',
        dest='motif_id_column',
        action='store',
        type=int,
        required=False,
        help='Specify which column contains the motif ID'
    )
    motif_group.add_argument(
        '-5',
        '--motif-md5-column',
        dest='motif_md5_column',
        action='store',
        type=int,
        required=False,
        help='Specify which column contains the motif md5 name'
    )

    parser.add_argument(
        '-c',
        '--columns',
        dest='one_based_column_indices_output',
        nargs='*',
        action='store',
        type=int,
        required=False,
        help='Specify which columns to use (default: all)'
    )
    parser.add_argument(
        '-r',
        '--rank',
        dest='display_rank',
        action='store_true',
        required=False,
        default=False,
        help='Display rank for each row in the HTML output.'
    )
    parser.add_argument(
        '-t',
        '--title',
        dest='title',
        action='store',
        type=str,
        required=False,
        help='Specify title which will be used in the HTML output.'
    )

    args = parser.parse_args()

    motif_to_tf_direct = MotifToTF.get_motif_to_tf(
        motif_to_tf_version=args.motif_collection_version,
        nomenclature=args.nomenclature,
        direct_or_indirect='direct',
    )
    motif_to_tf_indirect = MotifToTF.get_motif_to_tf(
        motif_to_tf_version=args.motif_collection_version,
        nomenclature=args.nomenclature,
        direct_or_indirect='indirect',
    )

    if args.motif_md5_column is not None:
        (motifs_count_md5_to_motif_id,
         motifs_count_md5_to_alternative_motif_ids) = MotifCollections.get_motifs_count_md5_to_motif_names(
            motif_collection_version=args.motif_collection_version
        )
    else:
        motifs_count_md5_to_motif_id = None
        motifs_count_md5_to_alternative_motif_ids = None

    convert_tsv_to_html(
        motifs_tsv_filename=args.tsv_filename,
        motifs_html_filename=args.html_filename,
        motif_to_tf_direct=motif_to_tf_direct,
        motif_to_tf_indirect=motif_to_tf_indirect,
        motif_collection_version=args.motif_collection_version,
        motif_id_column=(args.motif_id_column - 1 if args.motif_id_column is not None else None),
        motif_md5_column=(args.motif_md5_column - 1 if args.motif_md5_column is not None else None),
        motifs_count_md5_to_motif_id=motifs_count_md5_to_motif_id,
        motifs_count_md5_to_alternative_motif_ids=motifs_count_md5_to_alternative_motif_ids,
        column_indices_output=([one_based_column_idx - 1
                                for one_based_column_idx in args.one_based_column_indices_output]
                               if args.one_based_column_indices_output
                               else None),
        display_rank=args.display_rank,
        title=(args.title
               if args.title
               else os.path.basename(args.html_filename)),
    )

    sys.exit(0)


if __name__ == "__main__":
    main()
```
