# Supplementary material for: Cross-species analysis of enhancer logic using deep learning
Source: Genome Res. 2020 Dec;30(12):1815–34. doi: 10.1101/gr.260844.120 (PMC7706731; doi:10.1101/gr.260844.120)
Supplement: Supplemental Material [file supp_gr.260844.120_Supplemental_Code.zip › script_for_paper_DeepMEL_performance.html]

script\_for\_paper\_performance


# Notebook to measure the performance of DeepMEL¶

#### Following codes in this notebook were run using conda environments¶

#### Here are the used packages and their version¶

In [ ]:

```
%%bash
#cpu
conda create --name DeepMEL_conda_env_cpu python=3.6 tensorflow=1.14.0 keras=2.2.4
conda activate DeepMEL_conda_env_cpu
conda install numpy=1.16.2 matplotlib=3.1.1 shap=0.29.3 ipykernel=5.1.2

#gpu
conda create --name DeepMEL_conda_env_gpu python=3.6 tensorflow-gpu=1.14.0 keras-gpu=2.2.4
conda activate DeepMEL_conda_env_gpu
conda install numpy=1.16.2  matplotlib=3.1.1 shap=0.29.3 ipykernel=5.1.2
```

#### Loading necessary packages¶

In [1]:

```
import sys
import optparse
from array import *

import tensorflow
import numpy as np

import matplotlib
import matplotlib.pyplot as plt

import sklearn
from sklearn.utils import class_weight, shuffle
from sklearn.model_selection import train_test_split
from sklearn.metrics import average_precision_score, roc_auc_score

from keras.models import Sequential
from keras.models import model_from_json
from keras.layers.core import Dense, Dropout, Activation, Flatten
from keras.layers.convolutional import Conv1D, MaxPooling1D
from keras.layers import Bidirectional, Concatenate, PReLU 
from keras.optimizers import RMSprop, Adam
from keras.callbacks import ModelCheckpoint, EarlyStopping
from keras import regularizers
from keras.layers.wrappers import Bidirectional, TimeDistributed
from keras.layers.recurrent import LSTM
from keras.layers import Layer, average, Input
from keras.models import Model
from keras.utils import plot_model
```

```
Using TensorFlow backend.
```

#### Defining necessary functitons¶

In [2]:

```
def get_output(input_layer, hidden_layers):
    output = input_layer
    for hidden_layer in hidden_layers: 
        output = hidden_layer(output)
    return output

def build_model():
    forward_input = Input(shape=seq_shape)
    reverse_input = Input(shape=seq_shape)

    hidden_layers = [
        Conv1D(128, kernel_size=20, padding="valid", activation='relu', kernel_initializer='random_uniform'),
        MaxPooling1D(pool_size=10, strides=10, padding='valid'),
        Dropout(0.2),
        TimeDistributed(Dense(128, activation='relu')),
        Bidirectional(LSTM(128, dropout=0.1, recurrent_dropout=0.1, return_sequences=True)),
        Dropout(0.2),
        Flatten(),
        Dense(256, activation='relu'),
        Dropout(0.4),
        Dense(len(selected_classes), activation='sigmoid')]
    forward_output = get_output(forward_input, hidden_layers)     
    reverse_output = get_output(reverse_input, hidden_layers)
    output = average([forward_output, reverse_output])
    model = Model(input=[forward_input, reverse_input], output=output)

    model.summary()
    model.compile(optimizer='adam', loss='binary_crossentropy', metrics=['accuracy'])
    return model

def readfile(filename):
    ids = []
    ids_d = {}
    seqs = {}
    classes = {}
    f = open(filename, 'r')
    lines = f.readlines()
    f.close()
    seq = []
    for line in lines:
        if line[0] == '>':
            ids.append(line[1:].rstrip('\n'))
            if line[1:].rstrip('\n').split('_')[0] not in seqs:
                seqs[line[1:].rstrip('\n').split('_')[0]] = []
            if line[1:].rstrip('\n').split('_')[0] not in ids_d:
                ids_d[line[1:].rstrip('\n').split('_')[0]] = line[1:].rstrip('\n').split('_')[0]
            if line[1:].rstrip('\n').split('_')[0] not in classes:
                classes[line[1:].rstrip('\n').split('_')[0]] = np.zeros(NUM_CLASSES)
            classes[line[1:].rstrip('\n').split('_')[0]][int(line[1:].rstrip('\n').split('_')[1])-1] = 1        
            if seq != []: seqs[ids[-2].split('_')[0]]= ("".join(seq))
            seq = []
        else:
            seq.append(line.rstrip('\n').upper())
    if seq != []:
        seqs[ids[-1].split('_')[0]]=("".join(seq))
    return ids,ids_d,seqs,classes

def one_hot_encode_along_row_axis(sequence):
    to_return = np.zeros((1,len(sequence),4), dtype=np.int8)
    seq_to_one_hot_fill_in_array(zeros_array=to_return[0],
                                 sequence=sequence, one_hot_axis=1)
    return to_return

def seq_to_one_hot_fill_in_array(zeros_array, sequence, one_hot_axis):
    assert one_hot_axis==0 or one_hot_axis==1
    if (one_hot_axis==0):
        assert zeros_array.shape[1] == len(sequence)
    elif (one_hot_axis==1): 
        assert zeros_array.shape[0] == len(sequence)
    for (i,char) in enumerate(sequence):
        if (char=="A" or char=="a"):
            char_idx = 0
        elif (char=="C" or char=="c"):
            char_idx = 1
        elif (char=="G" or char=="g"):
            char_idx = 2
        elif (char=="T" or char=="t"):
            char_idx = 3
        elif (char=="N" or char=="n"):
            continue
        else:
            raise RuntimeError("Unsupported character: "+str(char))
        if (one_hot_axis==0):
            zeros_array[char_idx,i] = 1
        elif (one_hot_axis==1):
            zeros_array[i,char_idx] = 1

def create_plots(history):
    plt.plot(history.history['acc'])
    plt.plot(history.history['val_acc'])
    plt.title('model accuracy')
    plt.ylabel('accuracy')
    plt.xlabel('epoch')
    plt.legend(['train', 'test'], loc='upper left')
    plt.savefig(foldername + 'accuracy.png')
    plt.clf()

    plt.plot(history.history['loss'])
    plt.plot(history.history['val_loss'])
    plt.title('model loss')
    plt.ylabel('loss')
    plt.xlabel('epoch')
    plt.legend(['train', 'test'], loc='upper left')
    plt.savefig(foldername + 'loss.png')
    plt.clf()
    
def json_hdf5_to_model(json_filename, hdf5_filename):  
    with open(json_filename, 'r') as f:
        model = model_from_json(f.read())
    model.load_weights(hdf5_filename)
    return model

def loc_to_model_loss(loc):
    return json_hdf5_to_model(loc + 'model.json', loc + 'model_best_loss.hdf5')

def shuffle_label(label):
    for i in range(len(label.T)):
        label.T[i] = shuffle(label.T[i])
    return label

def calculate_roc_pr(score, label):
    output = np.zeros((len(label.T), 2))
    for i in range(len(label.T)):
        roc_ = roc_auc_score(label.T[i], score.T[i])
        pr_ = average_precision_score(label.T[i], score.T[i])
        output[i] = [roc_, pr_]
    return output
```

#### Preparing the input data¶

In [3]:

```
NUM_CLASSES = 24
selected_classes = np.array([1,2,3,4,5,6,7,8,9,10,11,12,13,14,15,16,17,18,19,20,21,22,23,24])-1
SEQ_LEN = 500
SEQ_DIM = 4
seq_shape = (SEQ_LEN, SEQ_DIM)

foldername = '/results/'
train_filename = '/summits_to_topics_wochr2.fa'
test_filename = '/summits_to_topics_chr2.fa'

print("Prepare input...")
train_ids, train_ids_d, train_seqs, train_classes = readfile(train_filename)
X_train = np.array([one_hot_encode_along_row_axis(train_seqs[id]) for id in train_ids_d]).squeeze(axis=1)
y_train = np.array([train_classes[id] for id in train_ids_d])
y_train = y_train[:,selected_classes]
X_train = X_train[y_train.sum(axis=1)>0]
y_train = y_train[y_train.sum(axis=1)>0]
X_train_rc = X_train[:,::-1,::-1]
train_data = [X_train, X_train_rc]

test_ids, test_ids_d, test_seqs, test_classes = readfile(test_filename)
X_test = np.array([one_hot_encode_along_row_axis(test_seqs[id]) for id in test_ids_d]).squeeze(axis=1)
y_test = np.array([test_classes[id] for id in test_ids_d])
y_test = y_test[:,selected_classes]
X_test = X_test[y_test.sum(axis=1)>0]
y_test = y_test[y_test.sum(axis=1)>0]
X_test_rc = X_test[:,::-1,::-1]
test_data = [X_test, X_test_rc]
```

```
Prepare input...
```

#### Loading the trained DeepMEL model¶

In [4]:

```
trained_model = loc_to_model_loss('/path_to_model/')
```

```
WARNING: Logging before flag parsing goes to stderr.
W0421 18:19:06.987329 47906138156672 deprecation_wrapper.py:119] From /staging/leuven/stg_00002/lcb/itask/programs/anaconda3/envs/deeplearning/lib/python3.6/site-packages/keras/backend/tensorflow_backend.py:517: The name tf.placeholder is deprecated. Please use tf.compat.v1.placeholder instead.

W0421 18:19:07.009386 47906138156672 deprecation_wrapper.py:119] From /staging/leuven/stg_00002/lcb/itask/programs/anaconda3/envs/deeplearning/lib/python3.6/site-packages/keras/backend/tensorflow_backend.py:74: The name tf.get_default_graph is deprecated. Please use tf.compat.v1.get_default_graph instead.

W0421 18:19:07.011919 47906138156672 deprecation_wrapper.py:119] From /staging/leuven/stg_00002/lcb/itask/programs/anaconda3/envs/deeplearning/lib/python3.6/site-packages/keras/backend/tensorflow_backend.py:4138: The name tf.random_uniform is deprecated. Please use tf.random.uniform instead.

W0421 18:19:07.038405 47906138156672 deprecation_wrapper.py:119] From /staging/leuven/stg_00002/lcb/itask/programs/anaconda3/envs/deeplearning/lib/python3.6/site-packages/keras/backend/tensorflow_backend.py:3976: The name tf.nn.max_pool is deprecated. Please use tf.nn.max_pool2d instead.

W0421 18:19:07.044372 47906138156672 deprecation_wrapper.py:119] From /staging/leuven/stg_00002/lcb/itask/programs/anaconda3/envs/deeplearning/lib/python3.6/site-packages/keras/backend/tensorflow_backend.py:133: The name tf.placeholder_with_default is deprecated. Please use tf.compat.v1.placeholder_with_default instead.

W0421 18:19:07.052615 47906138156672 deprecation.py:506] From /staging/leuven/stg_00002/lcb/itask/programs/anaconda3/envs/deeplearning/lib/python3.6/site-packages/keras/backend/tensorflow_backend.py:3445: calling dropout (from tensorflow.python.ops.nn_ops) with keep_prob is deprecated and will be removed in a future version.
Instructions for updating:
Please use `rate` instead of `keep_prob`. Rate should be set to `rate = 1 - keep_prob`.
W0421 18:19:08.984502 47906138156672 deprecation_wrapper.py:119] From /staging/leuven/stg_00002/lcb/itask/programs/anaconda3/envs/deeplearning/lib/python3.6/site-packages/keras/backend/tensorflow_backend.py:174: The name tf.get_default_session is deprecated. Please use tf.compat.v1.get_default_session instead.
```

#### Calculating auROC and auPR values¶

In [5]:

```
print('calculate roc and pr...')
roc_pr_dict = {"train": {}, "test": {}, "shuffle": {}}
roc_pr_dict["train"]["score"] = trained_model.predict(train_data)
roc_pr_dict["train"]["label"] = y_train
roc_pr_dict["test"]["score"] = trained_model.predict(test_data)
roc_pr_dict["test"]["label"] = y_test
roc_pr_dict["shuffle"]["score"] = np.array(roc_pr_dict["train"]["score"], copy=True)
roc_pr_dict["shuffle"]["label"] = shuffle_label(np.array(y_train, copy=True))

for sets in ["train", "test", "shuffle"]:
    roc_pr_dict[sets]["roc_pr"] = calculate_roc_pr(roc_pr_dict[sets]["score"], roc_pr_dict[sets]["label"])

fig = plt.figure(figsize=(25, 10))
ax = fig.add_subplot(2, 1, 1)
ax.set_ylabel('auROC')
ax.scatter(list(range(24)), roc_pr_dict["train"]["roc_pr"].T[0], color='red', label='TRAIN')
ax.scatter(list(range(24)), roc_pr_dict["test"]["roc_pr"].T[0], color='blue', label='TEST')
ax.scatter(list(range(24)), roc_pr_dict["shuffle"]["roc_pr"].T[0], color='gray', label='SHUFFLED')
ax.set_ylim([0, 1])
_ = plt.xticks(range(len(selected_classes)),range(1,len(selected_classes)+1))
ax.legend()

ax = fig.add_subplot(2, 1, 2)
ax.set_ylabel('auPR')
ax.scatter(list(range(24)), roc_pr_dict["train"]["roc_pr"].T[1], color='red', label='TRAIN')
ax.scatter(list(range(24)), roc_pr_dict["test"]["roc_pr"].T[1], color='blue', label='TEST')
ax.scatter(list(range(24)), roc_pr_dict["shuffle"]["roc_pr"].T[1], color='gray', label='SHUFFLED')
ax.set_ylim([0, 1])
_ = plt.xticks(range(len(selected_classes)),range(1,len(selected_classes)+1))
```

```
calculate roc and pr...
```
